# Pioneering the Future: Principles, Advances, and Challenges in Organic Electrodes for Aqueous Ammonium‐Ion Batteries

**DOI:** 10.1002/adma.202415676

**Published:** 2025-02-25

**Authors:** Mangmang Shi, Xiaoyan Zhang

**Affiliations:** ^1^ Department of Chemistry and Chemical Engineering Chalmers University of Technology Kemigården 4 Göteborg SE‐412 96 Sweden

**Keywords:** aqueous ammonium‐ion batteries, electrostatic interactions, hydrogen bonding, organic electrode materials

## Abstract

Aqueous ammonium‐ion (NH_4_^+^) batteries (AAIBs) have recently been considered as attractive alternatives for next‐generation large‐scale energy storage systems, on account of their cost‐effectiveness, nonflammability, less corrosive, small hydrated ionic radius, and rapid NH_4_^+^ diffusion kinetics. In addition, the tetrahedral structure of NH_4_^+^ exhibits preferential orientation characteristics, resulting in a different electrochemical storage mechanism from spherical charge carriers such as Li^+^, Na^+^, and K^+^. Therefore, unlocking the NH_4_^+^‐ion storage mechanisms in host electrode materials is pivotal to advancing the design of high‐performance AAIBs. Organic materials, with their customizable, flexible, and stable molecular structures, along with their ease of recycling and disposal, offer tremendous potential. However, the development of cutting‐edge organic electrode materials specifically for ammonium‐ion storage in AAIBs remains an exciting, yet largely untapped, frontier. This review systematically explores the interaction mechanisms between NH_4_^+^ ions and organic electrode materials, such as electrostatic interactions including hydrogen bonding. It also highlights the application of diverse organic electrode materials, such as small molecules, conducting polymers, covalent organic frameworks (COFs), and organic‐inorganic hybrids in AAIBs. Lastly, the review addresses the key challenges and future perspectives of organic‐material‐based AAIBs, aiming to push the boundaries of cutting‐edge aqueous energy storage systems.

## Graphical abstract

Leveraging the numerous advantages of ammonium‐ion (NH₄⁺)—including cost‐effectiveness, low corrosiveness, preferential orientation, and rapid diffusion kinetics—aqueous NH₄⁺ batteries (AAIBs) have gained significant attention. This review highlights and evaluates the progress of AAIBs utilizing organic electrode materials such as small molecules, conducting polymers, covalent organic frameworks, and organic‐inorganic hybrid materials, with the goal of advancing cutting‐edge aqueous energy storage systems.

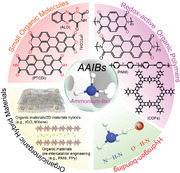

## 1 Introduction

Rechargeable batteries based on a mild aqueous electrolyte environment can fundamentally alleviate the safety concerns and flammability of organic‐electrolyte‐based batteries.^[^ ^1^ ^]^ Moreover, aqueous batteries (ABs) with fascinating features of cost‐effective, non‐toxicity, environmental benignity, facile fabrication, and ultrafast ionic transport capability, provide highly attractive alternatives for next‐generation high‐performance electrochemical energy storage/conversion devices, including large‐scale grid applications and future flexible wearable technologies.^[^ ^2^, ^3^, ^4^ ^]^ Actually, the development of ABs dates back to 1859, when Gaston Planté introduced lead‐acid batteries, using an aqueous H_2_SO_4_ solution as the electrolyte.^[^ ^5^, ^6^ ^]^ In 1994, Dahn's group was the first to demonstrate aqueous rechargeable lithium‐ion batteries (LIBs), showcasing their high safety and practicality by using a 5 M LiNO_3_ solution as the electrolyte.^[^ ^7^ ^]^ This research has since sparked a lot of research interest in ABs. Therefore, ABs based on univalent metal ions (e.g., Li^+^, Na^+^, and K^+^),^[^ ^8^, ^9^, ^10^ ^]^ multivalent metal ions (e.g., Zn^2+^, Mg^2+^, Ca^2+^ and Al^3+^),^[^ ^11^, ^12^, ^13^, ^14^ ^]^ non‐metallic cationic (e.g., H^+^, H_3_O^+^ and NH_4_^+^)^[^ ^15^, ^16^ ^]^ and non‐metallic anionic charge carriers (e.g., OH^‐^, F^‐^, Cl^‐^, I^‐^ and NO_3_^‐^)^[^ ^17^, ^18^, ^19^ ^]^ have generated numerous research findings and significant achievements in recent years.

**Figure 1:**
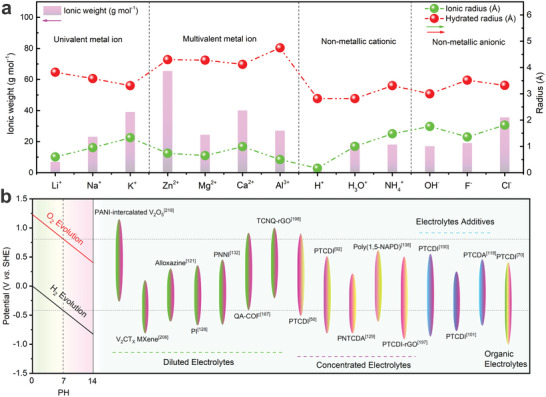
a) Comparison of ionic weight, ionic radius, and hydrated radius for typical charge carriers.^[^ ^1^ ^]^ b) Working windows of organic electrode materials for AAIBs.

Up to now, many research hotspots have shifted from Li^+^‐based ABs to those based on Na^+^, K^+^, and multivalent metal ions, by virtue of their low cost and greater natural abundance. In particular, multivalent metal ions as charge carriers can transfer more electrons during the insertion/extraction process and exhibit higher volumetric energy density, but the strong electrostatic interactions and the slow solid‐diffusion process hinder the further widespread usage of multivalent metal ion‐based ABs.^[^ ^20^, ^21^ ^]^ Compared to metal‐ion charge carriers, non‐metallic charge carriers, with the merits of resources abundant, favorable sustainability, relatively low molar mass, and smaller hydrated ion radius (**Figure** **1**a), provide new opportunities for inexpensive and state‐of‐the‐art ABs.^[^ ^19^ ^]^ Meanwhile, despite substantial achievements made in H^+^‐based aqueous proton batteries and OH^‐^‐based aqueous alkaline batteries,^[^ ^22^, ^23^, ^24^ ^]^ the strong acidic (e.g., H_2_SO_4_) and strong alkaline (e.g., KOH) electrolytes inevitably accelerate the corrosion of current collectors, electrode materials and devices, which undoubtedly raises system costs and poses challenges for large‐scale applications.

Fortunately, the hydrolysis of ammonium salts fosters a neutral to weakly acidic electrolyte environment due to the moderate acidity of NH_4_^+^.^[^ ^25^ ^]^ Even in highly concentrated NH_4_OAc solution (e.g., 30 m), the electrolyte maintains a slightly alkaline pH.^[^ ^26^ ^]^ Therefore, AAIBs with NH_4_^+^‐containing solution as electrolytes naturally mitigate the corrosion and side reactions.^[^ ^27^ ^]^ Meanwhile, resources abundant in NH_4_^+^ as charge carriers hold a relatively small hydrated ion radius of 3.31 Å and a lighter molar mass of ∼18 g mol^−1^, resulting in rapid diffusion kinetics of NH_4_^+^ in aqueous electrolyte.^[^ ^28^ ^]^ Additionally, unlike conventional spherical metal ions, NH_4_^+^ possesses a tetrahedral structure with the nitrogen atom as the center, showing preferential orientation characteristics.^[^ ^29^ ^]^ This also means that the intercalation/de‐intercalation electrochemical behavior of NH_4_^+^ charge carriers in host materials is different from that of spherical charge carriers. Therefore, uncovering the fascinating and unique topotactic insertion chemistry of NH_4_^+^ in host electrode materials is crucial for developing feasible and prospective AAIBs.^[^ ^30^ ^]^

Compared to metal ions, naked NH_4_^+^ exhibits a larger ionic radius (0.6 Å for Li^+^, 1.02 Å for Na^+^, 1.33 Å for K^+,^ and 1.48 Å for NH_4_^+^), making the selection of suitable host electrode materials more demanding and stringent.^[^ ^31^ ^]^ These host electrode materials need to provide more free spaces and a larger layer spacing for NH_4_^+^‐ion storage. Consequently, inorganic host electrode materials with 1D, 2D and 3D NH_4_^+^‐diffusion channels (e.g., α‐MnO_2_, VO_x_@polypyrrole (PPy) and Cu_0.4_Ni_1.6_Fe(CN)_6_) have emerged and achieved significant progress.^[^ ^32^, ^33^, ^34^ ^]^ During this period, organic host materials including 3,4,9,10‐perylenetetracarboxylic diimide (PTCDI), 1,4,5,8‐naphthalenetetracarboxylic dianhydride (NTCDA) and 3,4,9,10‐perylenetetracarboxylic dianhydride (PTCDA) have typically played a supporting role, complementing the rapidly advancing inorganic host materials in assembling NH_4_^+^‐ion full cells.^[^ ^35^ ^]^ Actually, organic electrode materials are very promising for NH_4_^+^‐ion storage, mainly because organic materials possess adjustable, flexible, and stable molecular structures, which can be conducive to accommodating the large ionic radius of NH_4_^+^.^[^ ^36^ ^]^ Despite the advancements in the field, there has yet to be a comprehensive review that systematically explores the structural engineering strategies and the mechanisms of NH_4_^+^ storage and transport in various organic electrodes for AAIBs.

In this review, we present a thorough overview of advancements in organic electrodes for AAIBs, focusing primarily on the selection of appropriate organic host electrode materials and examining the electrochemical behavior related to NH_4_^+^ storage in these electrodes. First of all, the details of the interaction mechanisms between NH_4_^+^ and organic electrode materials (electrostatic interactions including hydrogen bonding), are presented. Subsequently, the application of organic electrode materials such as small molecules, conducting polymers, COFs, and organic–inorganic hybrid materials in AAIBs, are systematically introduced. Meanwhile, in order to further improve the electrochemical performance of AAIBs, reasonably selecting matched organic electrodes with suitable operating windows and optimizing the electrolyte strategies are also discussed. Finally, the prospects and challenges of AAIBs that utilize organic materials, are highlighted. Given the growing interest in the development of novel AAIBs, it is both urgent and valuable to provide a comprehensive summary of AAIBs based on organic electrode materials.

## 2 Main Discussion

### 2.1 Operation Mechanism and Characterization Techniques of AAIBs

In fact, research on the electrochemical storage behavior of ammonium ions in Prussian Blue (PB) films began as early as 1982, using a 0.1 M NH_4_Cl solution as the NH_4_^+^ source.^[^ ^37^ ^]^ Subsequently, Cui's group reported that copper/nickel hexacyanoferrate (CuHCF and NiHCF) with open framework structures exhibited electrochemical activity in a 0.5 M (NH_4_)_2_SO_4_ solution.^[^ ^38^ ^]^ Besides, Gogotsi's group first reported the electrochemical intercalation of cations (including NH_4_^+^‐ions) into MXene in aqueous solution,^[^ ^39^ ^]^ which has also sparked research interest in NH_4_^+^ storage materials. So far, besides Prussian blue analogs (PBAs), significant efforts have been devoted to developing advanced electrode materials for high‐performance AAIBs, such as layered double hydroxides (LDHs), molybdenum‐based compounds, manganese‐based oxides, vanadium‐based oxides, and organic materials.^[^ ^40^ ^]^ In general, the electrochemical properties and NH_4_^+^ storage kinetics of individual electrode materials were initially assessed in a half‐cell configuration (three‐electrode electrochemical system) using an NH_4_^+^‐containing aqueous electrolyte. In this setup, the electrode materials serve as the working electrodes, Ag/AgCl or a saturated calomel electrode (SCE) acts as the reference electrode, and a carbon rod, carbon paper, or Pt plate functions as the counter electrode.^[^ ^41^, ^42^, ^43^ ^]^ As shown in Figure 1b, different electrode materials display distinct voltage windows when referenced against the standard hydrogen electrode (SHE). Organic electrode materials, which typically exhibit relatively low redox potentials, are often used as anodes in AAIBs. Some common inorganic compounds with high operating potential (e.g., PBAs, transition metal oxides) are usually employed as cathode materials to assemble full cell AAIBs. At present, similar to traditional metal‐ion batteries, most AAIBs still operate based on the “rocking‐chair” working principles, where NH_4_^+^‐ions as charge carriers migrate between the positive and negative electrodes during discharge and charge process. At this moment, NH_4_^+^‐ions are inserted/extracted into cathode and anode materials, via reversible hydrogen bond formation/breaking.^[^ ^44^ ^]^ Apparently, as the core components of AAIBs, electrode materials, and electrolyte directly determines the diffusion and intercalation/de‐intercalation behavior of NH_4_^+^‐ions, thereby affecting the electrochemical performance of AAIBs. Therefore, gaining an in‐depth understanding of NH₄⁺ storage and transport mechanisms through various characterization techniques is crucial for selecting suitable electrode materials and designing high‐performance AAIBs.

Until now various characterization techniques, such as in situ/ex situ X‐ray diffraction (XRD), Fourier‐transform infrared spectroscopy (FTIR), X‐ray photoelectron spectroscopy (XPS), scanning electron microscopy (SEM), and Raman spectroscopy, have been developed to monitor structural evolution of active materials, during the insertion/extraction process of charge carriers. It is necessary to combine several characterization methods to analyze the NH_4_^+^‐ion storage mechanism. For example, ex situ XRD characterizations at different states of charge (SOC) can uncover the reversibility of NH_4_^+^ intercalation in the lattice of hexagonal MoO_3_.^[^ ^45^ ^]^ Meanwhile, ex situ FTIR and solid‐state nuclear magnetic resonance (SSNMR) spectroscopy at different SOC can further confirm the reversible building‐breaking of HBs during the interaction between NH_4_^+^‐ions and host materials. More importantly, it is essential to confirm whether NH₄⁺ ions, rather than H⁺ ions or anions, are inserted into the electrode material. Ex‐situ characterization techniques can be employed to monitor the characteristic peaks of anions, thereby verifying their exclusion from the redox reaction.^[^ ^46^ ^]^ Organic electrode materials are typically tested in H₂SO₄ electrolyte with a pH identical to that of NH₄⁺‐containing electrolytes to evaluate the potential co‐intercalation of H⁺ with NH₄⁺. Additionally, in situ techniques such as XRD and FTIR have been employed to explore NH₄⁺ storage chemistry in detail.^[^ ^47^, ^48^ ^]^ Compared to ex‐situ measurements, in‐situ characterization techniques eliminate the need for additional workup processes, allowing for a more accurate reflection of the actual redox state of electrode materials. Therefore, the advancement and application of in‐situ testing techniques, such as in‐situ Raman spectroscopy, in‐situ NMR, and in‐situ SEM, are critically important for gaining a deeper understanding of the NH₄⁺ storage mechanism and enhancing the electrochemical performance of AAIBs.

Additionally, for both inorganic and organic electrode materials, density functional theory (DFT) calculations combined with experimental analyses, can provide deeper insights into the diffusion kinetics of NH₄⁺ ions within electrode materials and the formation of HBs between NH₄⁺ ions and host materials. For example, according to DFT calculations, α‐MnO_2_ (−5.07 eV) with the insertion of NH_4_^+^‐ions shows a lower adsorption energy than that of β‐MnO_2_ (9.58 eV) and γ‐MnO_2_ (1.64 eV), suggesting faster diffusion kinetics of NH_4_^+^ in α‐MnO_2_.^[^ ^49^ ^]^ Meanwhile, charge density distribution studies reveal that the formation of HBs between the NH_4_^+^‐ions and the α‐MnO_2_ can facilitate the movement of charge carriers. In another study, DFT calculations were conducted to explore the NH_4_^+^‐ion storage mechanism in the Fe_4_[Fe(CN)_6_]_3_ cathode.^[^ ^50^ ^]^ The results indicated that HBs formed between the hydrogen atoms of NH₄⁺ and the nitrogen atoms of Fe₄[Fe(CN)₆]₃ contribute to enhanced structural stability. As for organic electrode materials, the lowest unoccupied molecular orbital (LUMO), the highest occupied molecular orbital (HOMO), and their bandgap can be determined via theoretical calculations.^[^ ^51^ ^]^ For example, a π‐conjugated enhanced polyimide (PTPD) shows a narrow bandgap, indicating fast NH_4_^+^‐ion storage kinetics.^[^ ^52^ ^]^ Additionally, molecular electrostatic surface potential (MESP) analysis indicates that the C═O groups in the imide structures possess relatively negative potentials, facilitating the attraction of NH₄⁺ ions. Therefore, theoretical calculations can provide valuable insights into the structural evolution of electrode materials and the mechanism of NH₄⁺ ion uptake and removal.

### 2.2 Electrolyte Optimization of AAIBs

**Figure 2:**
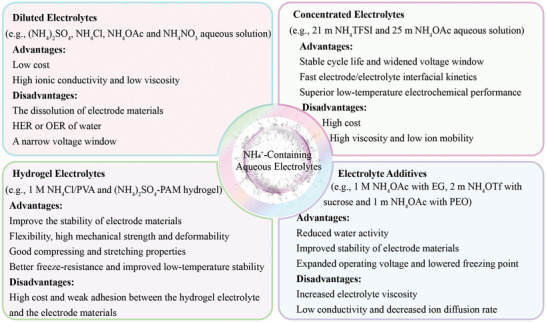
Advantages and disadvantages of NH_4_^+^‐containting aqueous electrolytes.

Moreover, the selection of electrolytes plays a crucial role in AAIBs, significantly affecting the NH_4_^+^‐ion storage behavior of electrode materials. The effectiveness of the electrolyte is determined by its solvation structure, viscosity, and ionic mobility, all of which impact the ionic conductivity and the electrochemical stability window of the system. Currently, a variety of NH_4_^+^‐containing electrolytes, including diluted electrolytes, hydrogel electrolytes, concentrated electrolytes, electrolyte additives, and even organic electrolytes, have been employed to investigate the NH_4_^+^‐ion storage chemistry of various electrode materials (**Figure** **2**).

Diluted electrolytes: Conventional diluted electrolytes primarily consist of ammonium salts, such as (NH_4_)_2_SO_4_, NH_4_Cl, NH_4_OAc, and NH_4_NO_3_, in aqueous solutions at low concentrations (e.g., 0.5 or 1 M). While these electrolytes offer low cost, fast ionic transport kinetics, and low viscosity, they still encounter several challenges, including parasitic hydrogen evolution reactions (HER) and oxygen evolution reactions (OER) occurring on the electrode surface. These reactions can lead to the dissolution or structural degradation of electrode materials and limit the overall voltage window.^[^ ^53^ ^]^ Additionally, the high activity of water molecules (acting as both H‐bond donors and acceptors) in diluted electrolytes promotes the formation of hydrogen bonding networks at temperatures below 0 °C, which limits their use in cold environments. Therefore, regulating the structure of diluted electrolytes via increasing the concentration and introducing additives can reconstruct or break the hydrogen‐bonding network, which can effectively expand the voltage window, suppress parasitic side reactions, and lower the freezing point, thereby improving the performance under cold conditions.^[^ ^54^ ^]^ Simultaneously, the flourishing development of AAIBs in diluted aqueous electrolytes has stimulated the research interest in their applications for next‐generation flexible wearable devices, due to their intrinsically non‐flammable nature, rapid ion transport capabilities, and low manufacturing costs.

Concentrated electrolytes: Concentrated electrolytes are produced by increasing the salt concentration in an aqueous solution. This approach significantly reduces the amount of free water molecules and limits their intercalation. As a result, concentrated electrolytes can minimize corrosion, broaden voltage window, and enhance the stability of electrode materials.^[^ ^55^ ^]^ Additionally, the absence of free water content will lower the freezing point of highly concentrated electrolytes, enhancing the low‐temperature performance of aqueous batteries.^[^ ^56^ ^]^ Liu's group demonstrated that the unique solvated NH_4_^+^ clusters formed in a highly concentrated electrolyte promote the adsorption and de‐solvation processes at the electrode/eletrolyte interface, leading to enhanced electrochemical performance.^[^ ^57^ ^]^ However, concentrated electrolytes inevitably raise the cost of battery assembly. Additionally, as the salt concentration increases, the viscosity of the electrolytes also rises, thereby limiting ion mobility.^[^ ^58^, ^59^ ^]^

Electrolyte additives: When introducing the electrolyte additives into NH_4_^+^‐containing electrolytes, the activity of water can be suppressed due to the formation of HBs between water and electrolyte additives, resulting in improved stability of electrode materials and expanded operating voltage.^[^ ^60^ ^]^ Furthermore, introducing organic solvents (e.g., acetonitrile, ethylene glycol) with low freezing points to create hybrid aqueous electrolytes can significantly reduce the freezing point of the electrolyte.^[^ ^61^ ^]^ However, adding a large amount of electrolyte additives may increase the viscosity and reduce the ionic conductivity of the electrolyte.^[^ ^62^ ^]^

Hydrogel electrolytes: Compared to liquid electrolytes, hydrogel electrolytes offer the advantages of solution retention and leakage prevention, rendering them crucial for quasi‐solid‐state, flexible, and stretchable energy storage devices.^[^ ^63^ ^]^ These electrolytes are typically composed of a hydrogel matrix, created by combining aqueous electrolytes with polymers like polyvinyl alcohol (PVA), sodium polyacrylate (PANa), or polyacrylamide (PAM). This unique structure allows for enhanced flexibility and performance in energy storage applications.^[^ ^64^, ^65^, ^66^ ^]^ Moreover, the rational design of hydrogel electrolytes can enable self‐healing and cold‐resistant aqueous energy storage devices.^[^ ^67^ ^]^ Notably, during the deformation process, strong adhesion is necessary to prevent delamination between the hydrogel electrolyte and the electrode materials.^[^ ^68^ ^]^

As research on optimizing aqueous electrolytes deepens, the aforementioned strategies are increasingly employed to further enhance the electrochemical performance of AAIBs. While much of the current research on NH_4_^+^‐ion storage focuses on aqueous environments, there is also progress being made in exploring the use of organic electrolytes. For instance, ammonium trifluoromethylsulfonate (NH_4_OTf) dissolved in succinonitrile (SN)^[^ ^69^ ^]^ and ammonium hexafluorophosphate (NH_4_PF_6_) dissolved in adiponitrile (ADN) or ethyl methyl carbonate (EMC) have been explored.^[^ ^70^ ^]^ Although organic electrolytes can effectively eliminate the intrinsic HER and OER and extend the potential window, their drawbacks such as flammability, toxicity, and low ionic conductivity,^[^ ^71^ ^]^ pose significant challenges for large‐scale applications. Overall, the combination of rational electrode material design with advanced electrolyte engineering offers a promising path for developing AAIBs with high energy density and a wide electrochemical window.

### 2.3 Hydrogen Bond Modulation and NH_4_^+^‐Ion Storage Mechanism in Organic Electrode Materials

The concept of HBs, a complex yet essential phenomenon, has undergone a long and evolving history of proposal and definition. The development of HBs can be traced back to the 1920s,^[^ ^72^ ^]^ which plays a vital role in the fields of biology, medicine, chemistry, and physics. The typical HB is described as X‐H···Y, where the X‐H is the proton donor and Y is the proton acceptor.^[^ ^73^ ^]^ Meanwhile, X is the high electronegative element (e.g., F, N, O) and Y is the proton acceptor.^[^ ^74^ ^]^ Furthermore, the X‐H bond exhibits a strong directional attraction to Y, and the formation of hydrogen bonding can greatly alter the physicochemical properties of the materials/molecules involved. In organic molecules, the presence of intermolecular or intramolecular HBs improves the stability of organic materials and facilitates charge carrier transport dynamics. For example, the formation of intermolecular HBs between the O atoms of the –C═O group and the H atoms of the ‐NH_2_ group in the 2,7‐diamino‐4,5,9,10‐tetraone (PTO‐NH_2_) molecule can effectively suppress the dissolution of PTO‐NH_2_ and improve its cycle stability.^[^ ^75^ ^]^ Similarly, the presence of intermolecular HBs among hydroxyl groups in the tetrahydroxy‐1,4‐benzoquinone disodium salt electrode materials enables good self‐healing properties and excellent cycle stability.^[^ ^76^ ^]^ Additionally, in the case of 1,2‐dihydroxyphenazine (PZ‐2OH), the introduction of hydroxyl groups can facilitate the formation of intramolecular/intermolecular HBs, which helps improve charge carrier transport and enhance redox kinetics.^[^ ^77^ ^]^ As a result, hydrogen bonding plays a crucial role in influencing electrochemical performance. Additionally, NH_4_^+^, with its four N─H groups serving as rich hydrogen bond donors, can form extensive hydrogen bonding interactions with organic electrode materials, largely affecting their behavior and performance.^[^ ^78^ ^]^

The stability of HBs is influenced by various factors, including the molecular environment, the nature of the participating atoms, and the surrounding physical conditions.^[^ ^79^ ^]^ The strength of HBs is significantly influenced by the electronegativity of the donor atom. Highly electronegative atoms, such as oxygen, nitrogen, or fluorine, enhance the partial positive charge on the attached hydrogen atom, thereby increasing the bond's overall strength. Conjugation or resonance within organic molecules or polymer systems can stabilize HBs by delocalizing charges across the structure. This effect enhances the overall stability of the hydrogen bonding network. Conjugated organic systems with strong HB sites, such as hydrogen‐bonded organic frameworks (HOFs), are being actively investigated for their potential to combine high structural stability with exceptional electrical conductivity. For example, HOF‐based structures have been used as the cathode for high‐performance aqueous batteries, showing exceptional long‐term capacity retention.^[^ ^80^, ^81^ ^]^ However, steric hindrance caused by bulky groups near the donor or acceptor sites can obstruct these interactions, diminishing the strength of the HBs. Additionally, during the discharge process, when NH₄⁺ ions interact with organic electrode materials, the HBs predominantly exhibit an ionic character due to the electrostatic attraction between the positive and negative charges. In AAIBs, the hydrogen‐bonding networks within the electrolytes primarily consist of H₂O‐H₂O and H₂O‐NH₄⁺ HBs. The diffusion of NH₄⁺ ions is heavily dependent on these established hydrogen‐bonding networks. HBs can promote NH₄⁺ hopping in aqueous electrolytes, facilitating ionic transport. However, a continuous hydrogen‐bonding network among water molecules can promote HER and OER reactions, rendering it unsuitable for high‐potential applications. To address this, the use of concentrated electrolytes or electrolyte additives can disrupt the hydrogen‐bonding network between water molecules, thereby mitigating HER and OER and expanding the voltage window.^[^ ^82^ ^]^ Additionally, HBs help stabilize water molecules, reducing the occurrence of unwanted side reactions in organic electrode materials. In aqueous environments, changes in pH can protonate or deprotonate donor and acceptor atoms, leading to significant alterations in hydrogen bonding interactions. In certain solid or gel electrolytes, a robust hydrogen‐bonding network can enhance mechanical stability (e.g., structural integrity and flexibility) and mitigate solvent evaporation or leakage.

**Figure 3:**
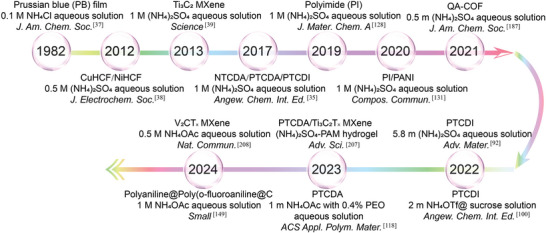
Time line of organic materials in AAIBs, including copper hexacyanoferrate (CuHCF), nickel hexacyanoferrate (NiHCF), polyaniline (PANI), 3,4,9,10‐perylenetetracarboxylic diimide (PTCDI), 1,4,5,8‐naphthalenetetracarboxylic dianhydride (NTCDA) and 3,4,9,10‐perylenetetracarboxylic dianhydride (PTCDA), COFs based on quinone carbonyl oxygen and pyrazine nitrogen monomer units (QA‐COF), ammonium trifluoromethanesulfonate (NH_4_OTf) and poly(ethylene oxide) (PEO).

**Figure 4:**
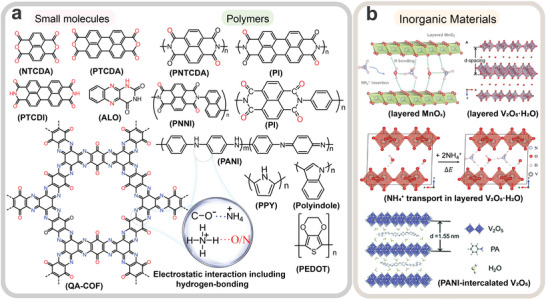
a) Schematic diagram of typical organic compounds for NH_4_^+^‐ion storage. b) Inorganic materials including layered MnO_x_, layered V_2_O_5_·H_2_O, and PANI‐intercalated V_2_O_5_ for AAIBs. b) Layered MnO_x_ (Top): Reproduced with permission.^[^ ^85^ ^]^ Copyright 2020, Wiley‐VCH. Layered V_2_O_5_⋅H_2_O (Top and Middle): Reproduced with permission.^[^ ^86^ ^]^ Copyright 2019, Elsevier B.V. PANI‐intercalated V_2_O_5_ (Bottom): Reproduced with permission.^[^ ^47^ ^]^ Copyright 2022, Royal Society of Chemistry.

Organic electrode materials are critical to the function of AAIBs, as they govern the insertion and extraction of NH_4_^+^‐ions, which in turn has a direct impact on the overall electrochemical performance of AAIBs. In 2017, the research into the NH_4_^+^‐ion storage behavior of aromatic compounds such as PTCDI, NTCDA, and PTCDA sparked interest in using organic electrode materials for high‐performance AAIBs (**Figure** **3**).^[^ ^35^ ^]^ Subsequently, a series of polymers and organic/inorganic hybrid materials derived from aromatic compounds were used for NH_4_^+^‐ion storage. **Figure** **4**a shows the typical chemical structures of different organic compounds. In situ/ex situ FTIR and theoretical calculations have been proven powerful tools for characterizing HBs. These organic compounds possess rich C═O or C═N functional groups, which serve as redox‐active sites capable of accommodating NH₄⁺ ions through electrostatic interactions. For example, they can form N‐H···O and N‐H···N HBs with oxygen or nitrogen atoms. Typically, in order to clearly characterize the NH_4_^+^‐ion storage chemistry in the PTPD, in situ FT‐IR analysis measurements were performed.^[^ ^52^ ^]^ In situ FT‐IR analysis reveals that during the charging process (when NH₄⁺ ions are released), the characteristic peak of the C═O bond and O═C─N bond (1361 cm⁻¹) intensifies, while the peak of the C─O bond and O─C─N bond (1323 cm⁻¹) diminishes. Conversely, these changes reverse during the discharge process, confirming the reversible insertion and extraction of NH₄⁺ ions within the PTPD framework. Notably, ex situ FTIR spectra of 2,7‐dinitropyrene‐4,5,9,10‐tetraone (DNPT) indicate not only the transformation from the C═O group (1691 cm^−1^) to C─O group (1421 cm^−1^), but also the emergence of a new C─O···NH_4_ HB (2845 cm^−1^) during the discharge process^[^ ^83^ ^]^ Meanwhile, another new characteristic peak at 2922 cm^−1^ is attributed to the N─H bonds of NH_4_^+^‐ions, further confirming the intercalation of NH_4_^+^‐ions during the discharge process. In addition, as for the N‐containing organic compounds (aza‐based COF), the results of ex situ FTIR show the reversible transformation from the C = N bond to the C‐N bond during the discharge/charge process.^[^ ^84^ ^]^ Besides, the characteristic peak of N‐H bonds including the N‐H bonds of NH_4_^+^‐ions and the N‐H···N HBs (≈3000 cm^−1^) also reversibly change, confirming the formation of HBs between NH_4_^+^‐ions and the N atoms. Furthermore, DFT simulation results indicate that the formed N─H···N HBs are weak in contrast to ionic‐covalent bonds and metal‐coordinated bonds, endowing the rapid ion diffusion kinetics. The effect of π–π stacking and HBs can be further verified through aggregation‐induced changes in UV–vis spectroscopy.^[^ ^80^ ^]^ DFT simulations including excited‐state intramolecular proton transfer (ESIPT) and gradient isosurface can also indicate the formation of HBs. In addition, NMR and Raman spectroscopy can be used as supplementary techniques to confirm the formation of HBs, as evidenced by shifts of relevant NMR peaks and Raman bands.

In addition, the diffusion of NH_4_^+^‐ions across electrode materials and electrolytes relies on the continuous formation and breaking of HBs.^[^ ^84^ ^]^ Organic molecules containing electronegative atoms such as N, O, and F can form hydrogen bonds with NH_4_^+^ charge carriers, thereby reducing the diffusion barrier for NH_4_^+^ ions. For example, when NH_4_^+^‐ions are present in the layers or tunnels of inorganic compounds (e.g., layered MnO_x_,^[^ ^85^ ^]^ Layered V_2_O_5_·H_2_O^[^ ^86^ ^]^ or PANI‐intercalated V_2_O_5_^[^ ^47^ ^]^) (Figure 4b), they can also form HBs with the O atoms (HB acceptors). Here, unlike the migration of spherical metal ions, the diffusion of NH_4_^+^‐ions undergoes twisting and rotation to break and reform HBs, which can improve the NH_4_^+^‐ions diffusion kinetics inside the electrode material.^[^ ^87^ ^]^ However, with the repeated insertion and extraction of NH_4_^+^, the open structure of inorganic compounds will collapse due to lattice expansion and contraction, resulting in capacity fading. In contrast, the NH_4_^+^‐ions are mainly adsorbed on the redox‐active groups of organic materials (e.g., C═O, C═N), which are different from the intercalation‐type mechanism of inorganic materials. However, when organic materials, in particular small organic molecules, are combined with 2D materials such as graphene, they can anchor to the graphene surface, enabling redox reactions to predominantly occur on the surface of the hybrid material. In some cases, organic molecules retain a well‐defined crystal structure, such as PTO‐NH_2_ and PTCDA molecules.^[^ ^75^, ^88^ ^]^ Consequently, the diffusion of NH₄⁺ ions within these organic materials remains a critical area of interest requiring further investigation. Moreover, organic materials offer more flexible structures, adjustable internal voids, and abundant exposed surface sites, rendering them highly promising for NH_4_^+^‐ion storage.

### 2.4 Organic Electrode Materials for AAIBs

#### 2.4.1 Small Organic Molecules

##### AAIBs Based on PTCDI Anode

Currently, a variety of inorganic materials have been developed and proven to show excellent electrochemical performance as cathodes for AAIBs. In the case of NH_4_^+^‐ion full cells, the anode is equally critical, as it directly influences the full cell's capacity, rate capability, and lifespan. However, research and interest in anode materials remain relatively underexplored. To date, several inorganic materials (e.g., α‐MoO_3_/Ti_3_C_2_T_z_, hexagonal MoO_3_, monoclinic WO_3_) with a low potential, have been utilized as anodes for AAIBs.^[^ ^45^, ^89^, ^90^ ^]^ However, compared to inorganic options, many organic materials are considered more suitable as anodes for AAIBs due to their lower potential, offering promising advantages for improved battery performance.^[^ ^25^ ^]^

**Figure 5:**
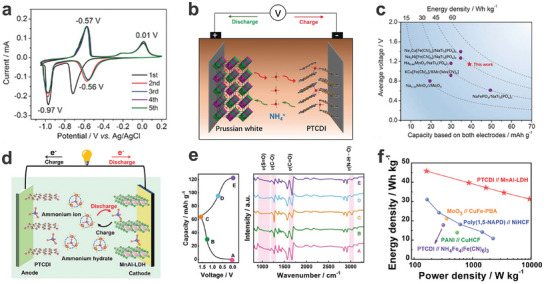
a) The CV curves of PTCDI at 0.5 mV s^−1^ in a 1 M (NH_4_)_2_SO_4_ electrolyte. b) Schematic illustration of the rocking‐chair Ni‐APW//PTCDI AAIB. c) A comparison of the electrochemical performance with other batteries. a‐c) Reproduced with permission.^[^ ^35^ ^]^ Copyright 2017, Wiley‐VCH.d) Schematic illustration of the MnAl‐LDH//PTCDI full cell in a 1 M (NH_4_)_2_SO_4_ electrolyte. e) GCD curves and the corresponding ex situ FT‐IR spectra, f) Ragone plot of the MnAl‐LDH//PTCDI full cell. d‐f) Reproduced with permission.^[^ ^46^ ^]^ Copyright 2022, Wiley‐VCH.

As a typical organic anode material, PTCDI was first reported by Ji's group in a 1 M (NH_4_)_2_SO_4_ aqueous electrolyte.^[^ ^35^ ^]^ Actually, three aromatic compounds, such as NTCDA, PTCDA, and PTCDI were explored for NH_4_^+^‐ion storage. Notably, while NTCDA and PTCDA exhibit significantly higher capacities during the first cycle compared to PTCDI, their cycling stability is considerably lower. This decreased stability may be linked to the solubility of intermediate compounds and/or phase transition processes. However, the exact mechanism behind this capacity fading remains unclear and requires further investigation. As shown in **Figure** **5**a, the shape of the CV curves for the PTCDI anode in a 1 M (NH_4_)_2_SO_4_ electrolyte shows good preservation after the first cathodic process, demonstrating the high reversibility of the PTCDI anode for NH_4_^+^‐ion storage during the charge/discharge process. The PTCDI anode delivered a high capacity of 158.9 mAh g^−1^ at a current density of 0.24 A g^−1^ in the first cycle and maintains a high cycle stability, due to its high electrochemical activity, steady π‐conjugated structure and low solubility in aqueous solution. The as‐fabricated Ni‐APW((NH_4_)_1.47_Ni[Fe(CN)_6_]_0.88_)//PTCDI full cell (Figure 5b) showed a specific capacity of 41 mAh g^−1^ at a current density of 0.06 A g^−1^ and an excellent energy density of ≈43 Wh kg^−1^ (based on the total active mass loading of both the anode and cathode) (Figure 5c). As a result, the successful application of PTCDI has led to its widespread use as an organic anode material in AAIBs. For example, Shu's group successfully fabricated a rocking‐chair aqueous NH_4_^+^ full cell with PTCDI as the anode in saturated (NH_4_)_2_SO_4_, where NH_4_·Fe_4_[Fe(CN)_6_]_3_ was used as the cathode.^[^ ^50^ ^]^ The NH_4_·Fe_4_[Fe(CN)_6_]_3_//PTCDI full cell exhibits cycling stability of 89.8% after 300 cycles. However, an extended cycling stability test is necessary to provide a more reliable demonstration of the cell's long‐term performance. Besides using PBAs as the cathode, Hu's group employed Mn‐Al layered double hydroxide (MnAl‐LDH) as the cathode to fabricate a MnAl‐LDH//PTCDI full cell in a 0.5 M (NH_4_)_2_SO_4_ electrolyte (Figure 5d).^[^ ^46^ ^]^ The GCD curves of the full cell are displayed in Figure 5e, showing a specific capacity of 57.7 mAh g^−1^ at a current density of 0.1 A g^−1^ based on the total active mass loading of both the anode and cathode. As displayed in the ex situ FT‐IR spectra of the PTCDI anode, the intensity of the carbonyl groups (C═O) first decreases and then recovers during the charge and discharge process, indicating the reversible evolution of the carbonyl groups in the PTCDI anode. Besides, the characteristic absorption peaks of the S═O group (≈1000 cm^−1^) can hardly be observed at any state of charge, implying that the NH_4_^+^ cation as charge carriers participates in the energy storage process rather than the SO_4_^2‐^ group. Ultimately, the assembled MnAl‐LDH//PTCDI full cell showed a maximum energy density of 45.8 Wh kg^−1^ (Figure 5f).

Inspired by the aforementioned attractive NH_4_^+^‐ion storage chemistry of PTCDI in aqueous electrolytes, a series of NH_4_^+^‐ion energy storage systems based on PTCDI anodes have been developed and designed in a different type of NH_4_^+^‐containing electrolytes (e.g., hydrogel electrolytes,^[^ ^91^ ^]^ concentrated electrolytes,^[^ ^92^ ^]^ and organic electrolytes^[^ ^93^ ^]^). For instance, Zhang's group reported a flexible quasi‐solid‐state NH_4_^+^‐ion storage device with NH_4_Cl/PVA hydrogel as the electrolyte, where the polymer‐intercalated vanadium oxide hydrate coated on activated carbon cloth (denoted as ACC@VPP) and PTCDI were used as the cathode and anode, respectively.^[^ ^91^ ^]^ The ACC@VPP//PTCDI NH_4_^+^‐ion storage device displayed outstanding flexibility, providing great potential for practical applications in flexible wearable energy devices. Subsequently, the same group successfully fabricated an Od‐NHVO//PICDI NH_4_^+^‐ion storage device in a 1 M NH_4_Cl/PVA hydrogel electrolyte, using ammonium vanadate ((NH_4_)_2_V_10_O_25_·8H_2_O) with oxygen defects (denoted as Od‐NHVO) as the cathode and PTCDI as the anode. The full cell not only showed reliable flexibility but also demonstrated a remarkable area energy density of 3 Wh m^−2^.^[^ ^94^ ^]^ However, conventional PVA‐based hydrogel electrolytes tend to lose water during the charge/discharge process, leading to decreased cycling stability. To address this issue, it is crucial to develop new flexible polymer hydrogels with enhanced electrolyte retention.

Passerini's group reported the fabrication of AAIBs in a concentrated electrolyte (5.8 M (NH_4_)_2_SO_4_), where ammonium copper hexacyanoferrate (N‐CuHCF) and PTCDI were used as the cathode and anode, respectively.^[^ ^92^ ^]^ The comparison of GCD results for the PTCDI anode in a diluted 1 M (NH_4_)_2_SO_4_ electrolyte (denoted as LCE) and a 5.8 M (NH_4_)_2_SO_4_ electrolyte (denoted as HCE) demonstrated more stable long‐term cycling stability in HCE (≈98% of capacitance retention after 5000 cycles), ascribed to the less dissolution of the PTCDI anode in HCE. Additionally, the N‐CuHCF//PTCDI full cell exhibited a specific capacity of 48.2 mAh g^−1^ based on the active mass loading of the cathode, with 72% capacity retention after 1000 cycles. Besides, the reported A‐PBA ((NH_4_)_1.85_Fe_0.33_Mn_0.67_[Fe(CN)_6_]_0.98_·0.77H_2_O)//PTCDI fell cell in 21 m NH_4_TFSI concentrated aqueous electrolyte showed excellent cycling stability and energy density.^[^ ^95^ ^]^ Importantly, trifluoromethane sulfonate (OTf^‐^)‐based electrolytes, such as zinc trifluoromethanesulfonate Zn(OTf)_2_,^[^ ^96^ ^]^ have garnered significant attention due to their unique chemical structure. The OTf⁻ anion consists of both a hydrophobic (‐CF_3_) and a hydrophilic (‐SO_3_⁻) group, offering distinct advantages in electrolyte performance.^[^ ^97^ ^]^ Unlike the hydrophilic inorganic SO_4_^2−^ anion, the OTf^‐^ anion can interact with both water and various common organic molecules (e.g., propylene carbonate, dimethyl carbonate), endowing trifluoromethane sulfonate with fascinating chemical properties. For example, Niu's group reported that Fe‐substituted manganese‐based Prussian blue analog (FeMnHCF) cathodes work exceptionally well with a highly concentrated electrolyte (24 m NH_4_CF_3_SO_3_).^[^ ^98^ ^]^ The concentrated electrolytes can effectively suppress the activity of water molecules, owing to the reconstruction of hydrogen bonding networks, which can ensure the high‐potential reversibility of the FeMnHCF cathode and restrain the dissolution of electrode materials. Meanwhile, the low‐hydration architecture of NH_4_^+^ in concentrated electrolytes is conducive to the rapid NH_4_^+^‐ion storage kinetics in host electrode materials. The as‐assembled FeMnHCF//PTCDI full cell in a 24 m NH_4_CF_3_SO_3_ electrolyte showed a specific capacity of 123.8 mAh g^−1^ at a current density of 0.5 A g^−1^ and a high energy density of ≈71 Wh kg^−1^ based on the total active mass loading of both the anode and cathode. Apparently, NH_4_^+^ can effectively improve the electrochemical performance of PBAs, attributed to that NH_4_^+^ tends to be inserted into the voids of PBAs in the form of naked ions.

**Figure 6:**
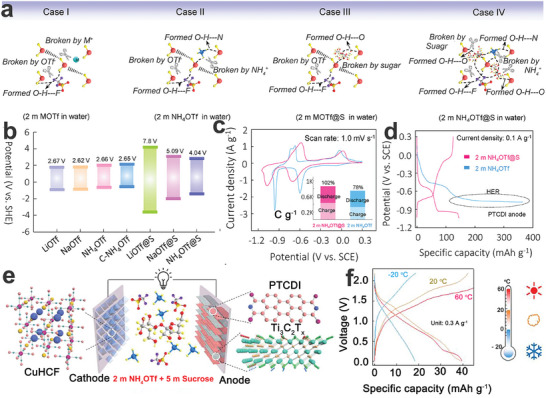
a) Comparison of four different electrolyte models. b) Overpotential variations for electrolytes. c) CV curves, d) and GCD curves of the PTCDI anode in a 2 m NH_4_OTf electrolyte and a 2 m NH_4_OTf@S electrolyte. e) Illustration of the CuHCF//PTCDI@MXene full cell. f) GCD curves under different temperatures. a–f) Reproduced with permission.^[^ ^100^ ^]^ Copyright 2022, Wiley‐VCH.

In addition to constructing concentrated electrolytes and weakly solvated electrolytes, the introduction of electrolyte additives is a very effective strategy for improving the electrochemical performance of energy storage systems.^[^ ^99^ ^]^ Alshareef's group proposed an H‐bond modulation strategy by adding an electrolyte additive (sucrose) into an aqueous ammonium sulfonate solution (NH_4_OTf), where the hydroxyl‐rich sucrose molecules can interact with water molecules via HBs, disrupting the original water H‐bonding network.^[^ ^100^ ^]^ Therefore, the optimized electrolyte can significantly suppress the decomposition of water and the dissolution of the electrode materials, enabling the development of an ultra‐stable NH_4_^+^‐ion full battery. Specifically, to illustrate the effect of the sucrose electrolyte additive on the H‐bonding networks, the comparison of four different electrolyte models is displayed in **Figure** **6**a, including metal salts (2 m MOTf, Case I), metal salts with sucrose (2 m MOTf@S, Case III), ammonium salts (2 M NH_4_OTf, Case II) and ammonium salts with sucrose (2 m NH_4_OTf@S, Case IV). Unlike metal cations, NH_4_^+^ can help restore the disrupted hydrogen bonding network of water. However, the addition of sucrose restructures this network by forming sucrose‐water HBs, thereby breaking the original water hydrogen‐bonding network. As shown in Figure 6b, electrolytes without sucrose exhibit more pronounced HER and OER, most likely due to the intact water hydrogen bonding network. In contrast, NH_4_^+^ migrates more efficiently along the sucrose‐water hydrogen bonding networks, facilitated by the formation of weak HBs between NH_4_^+^ and sucrose. Subsequently, the CV curves of the PTCDI anode (Figure 6c) were recorded in a 2 m NH_4_OTf (0.3 to −1.0 V) electrolyte and a 2 m NH_4_OTf@S (0.3 to −1.1 V) electrolyte, demonstrating that the PTCDI anode possessed a more negative HER potential in the 2 m NH_4_OTf@S electrolyte. Additionally, compared to the PTCDI anode in the 2 m NH₄OTf@S electrolyte, the PTCDI anode in the 2 m NH₄OTf electrolyte exhibited significant side reactions. Furthermore, as shown in Figure 6d, the PTCDI anode even cannot discharge normally in the 2 m NH_4_OTf electrolyte due to the severe HER process. In contrast, the PTCDI anode delivered a high specific capacity of 145 mAh g^−1^ at a current density of 0.1 A g^−1^ without obvious side reactions in the 2 m NH_4_OTf@S electrolyte. The assembled copper hexacyanoferrate (CuHCF)//PTCDI@MXene full cell (Figure 6e) in a 2 m NH_4_OTf@S electrolyte showed a stable operating voltage window (0 to 2.2 V) and showcased a high specific capacity of 41 mAh g^−1^ at a current density of 0.2 A g^−1^ (20 °C) based on the total active mass loading of both the anode and cathode. Interestingly, the CuHCF//PTCDI@MXene full cell showed good electrochemical performance (Figure 6f) under different working temperatures (‐20, 20, and 60 °C). Similarly, as a typical electrolyte additive, ethylene glycol (EG) with rich hydroxyl groups can reconstruct the water H‐bonding network via the formation of water‐EG HBs with water molecules, which favors lowering the water activity, broadens the working potential window and reduces the dissolution of electrode materials.^[^ ^101^ ^]^ Therefore, the PTCDI anode showed great electrochemical reversibility and a high capacity of 77.9 mAh g^−1^ in a 1 M NH_4_OAc electrolyte mixed with 40 v/v% EG (EG‐40). Due to the serious HER side reaction, the PTCDI anode cannot discharge in a 1 M NH_4_OAc electrolyte. The as‐farbicated CuHCF//PTCDI full cell in EG‐40 delivered specific capacity retention of 82.8% after 500 cycles, as well as an energy density of 63.1 Wh kg^−1^ at a power density of 262.7 W kg^−1^ based on the active mass loading of the cathode. Furthermore, EG as a well‐known antifreeze agent is expected to significantly improve the low‐temperature electrochemical performance of the NH_4_^+^‐ion full cell and broaden its operating temperature.^[^ ^102^ ^]^ Recently, Varzi's group used EG as a hydrogen‐bonding modulator and antifreeze agent to enhance the stability and low‐temperature performance of AAIBs.^[^ ^60^ ^]^ With the introduction of EG into a 1 m NH_4_OAc electrolyte (H_2_O:EG = 5:5, volume ratio), the PTCDI anode delivered improved electrochemical stability and showed capacity retention of 64% after 5000 cycles. Besides, the full NH_4_^+^‐ion battery demonstrated a discharge capacity of 61 mAh g^−1^ at ‐20 °C. Additionally, other reported electrolyte additives (e.g., poly(ethylene glycol), glycerol)^[^ ^103^ ^]^ can also be explored to implement ultra‐stable and high‐voltage AAIBs.

**Figure 7:**
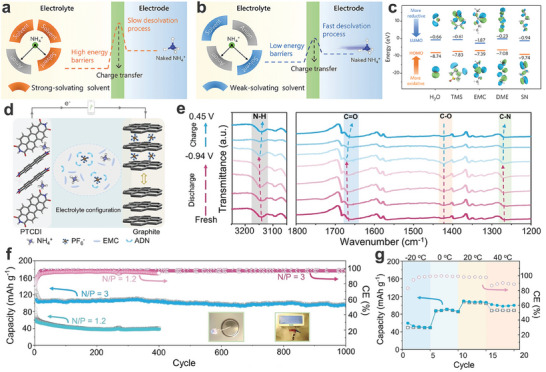
Desolvation kinetics of NH_4_^+^ in electrolytes with a) a strong‐solvating solvent, b) and weak‐solvating solvent. c) The LUMO and HOMO energy levels. a‐c) Reproduced with permission.^[^ ^69^ ^]^ Copyright 2023, Wiley‐VCH. d) Illustration of the graphite//PTCDI organic NH_4_^+^‐ion full cell. e) Ex situ FT‐IR analysis for the PTCDI anode. f) Cycling performance, and g) electrochemical performance at varied temperatures of the graphite//PTCDI full cell. d‐g) Reproduced with permission.^[^ ^70^ ^]^ Copyright 2022, Wiley‐VCH.

In comparison, there have been a few reports on organic NH_4_^+^‐containing electrolytes. Specifically, the desolvation energy of NH_4_^+^ is primarily determined by its binding energy and the interaction ability with solvents, as well as the number of solvent molecules involved. The simple schematic diagram is described to show the comparison of desolvation kinetics of NH_4_^+^ in strong‐solvating solvents (**Figure** **7**a) and weak‐solvating solvents (Figure 7b).^[^ ^69^ ^]^ Notably, in electrolytes with strongly interacting solvents, these solvents preferentially occupy many of the interaction sites with NH₄⁺. In contrast, in electrolytes with weakly interacting solvents, more anions are able to interact with NH₄⁺, encouraging the formation of contact ion pairs or ion aggregates. This may result in a lower desolvation energy barrier and a faster desolvation process. In this work, weakly solvated NH_4_^+^ electrolytes were designed by mixing ammonium trifluoromethylsulfonate (NH_4_OTf) with succinonitrile (SN). The calculated electrostatic potential (ESP) results show that SN exhibited the highest ESP of −30.9 kcal mol^−1^, compared to the ESP values of tetramethylene sulfone (TMS, −45.2 kcal mol^−1^), ethyl methyl carbonate (EMC, −36.5 kcal mol^−1^), dimethoxy ethane (DME, −35.3 kcal mol^−1^), and H_2_O (−37.3 kcal mol^−1^). Therefore, SN as a solvent possibly possesses a weaker solvation ability. Additionally, the energy levels of the LUMO and HOMO for H_2_O, TMS, EMC, DME, and SN were calculated (Figure 7c). Obviously, SN exhibited the lowest LUMO/HOMO energy levels (−0.94/−9.74 eV), implying its strong resistance to reduction and antioxidant capability, which is beneficial for the expansion of the electrochemical window of electrolytes. Finally, the full cell was assembled by employing NH_4_^+^ Mn‐rich PBAs (A‐MnPBA, (NH_4_)_1.81_Fe_0.28_Mn_0.72_[Fe(CN)_6_]_0.96_·0.85H_2_O) as the cathode and PTCDI as the anode, and a 2.5 m NH_4_OTf in SN as the electrolyte. The A‐MnPBA//PTCDI full cell was dominated by the pseudocapacitive effect, showing a reversible capacity of 116 mAh g^−1^ at a current density of 0.5 A g^−1^ based on the active mass loading of the cathode. Similarly, a rocking‐chair VOPO_4_·2H_2_O//PTCDI full cell was assembled using a 2 M NH_4_OTf acetonitrile solution as the electrolyte, delivering a specific capacity of 55 mAh g^−1^ at a current density of 0.1 A g^−1^.^[^ ^104^ ^]^ This strategy of constructing weakly solvated electrolytes provides an opportunity to improve the electrochemical performance of organic electrode materials. Unfortunately, the impact of varying electrolyte concentrations on the PTCDI anode has not been thoroughly explored, underscoring the need for more focused research in the development of anodes for organic‐electrolyte‐based AIBs. Meanwhile, a Prussian white analog (MnHCF)//PTCDI organic NH_4_^+^‐ion full cell,^[^ ^93^ ^]^ with a 1 M bis(trifluoromethane)sulfonimide ammonium (NH_4_TFSI) in tetraethylene glycol dimethyl ether (TEGDME) as the electrolyte, delivered a relatively high output voltage platform of ≈1.5 V and a broad electrochemical window. Notably, the PTCDI anode exhibited poor electrochemical performance in organic electrolytes, resulting in unsatisfactory energy density and cycling stability of organic NH_4_^+^‐ion full cells. Therefore, developing high‐performance organic anodes and well‐matched organic electrolytes is both urgent and essential. As a proof‐of‐concept, Alshareef's group reported a NH_4_^+^‐based dual‐ion battery in organic electrolytes by employing PTCDI as the anode and graphite as the cathode (Figure 7d).^[^ ^70^ ^]^ The organic electrolyte is composed of 1 m ammonium hexafluorophosphate (NH_4_PF_6_) in a mixed solvent of adiponitrile (ADN, anti‐oxidative molecules) and ethyl methyl carbonate (EMC, reduction‐resistant molecules), forming a 1 m NH_4_PF_6_/ADN‐EMC solution. This electrolyte can simultaneously provide NH_4_^+^ cations and PF_6_^‐^ anions as charge carriers while delivering a wide electrochemical window of 4.5 V. Ex situ FT‐IR analysis for the PTCDI anode was carried out in the NH_4_PF_6_/ADN‐EMC electrolyte to reveal the NH_4_^+^‐ion storage mechanism (Figure 7e). With the insertion of NH_4_^+^ into the PTCDI anode, the carbonyl groups (C═O, ≈1658 cm^−1^) gradually evolved into enolate groups (C═O, ≈1422 cm^−1^). Conversely, the intensity of these peaks returned to their original state, demonstrating the high reversibility of the NH_4_^+^ insertion/extraction process. The optimized graphite//PTCDI full cell featured a high operating voltage of 2.75 V and excellent durability, retaining 88% of its capacity after 1000 cycles (Figure 7f). Besides, the graphite//PTCDI full cell showed normal operation even at −20 °C (49.8 mAh g^−1^) (Figure 7g). This strategy provides a feasible path for achieving high‐voltage NH_4_^+^ full cells.

##### AAIBs Based on PTCDA Anode

**Figure 8:**
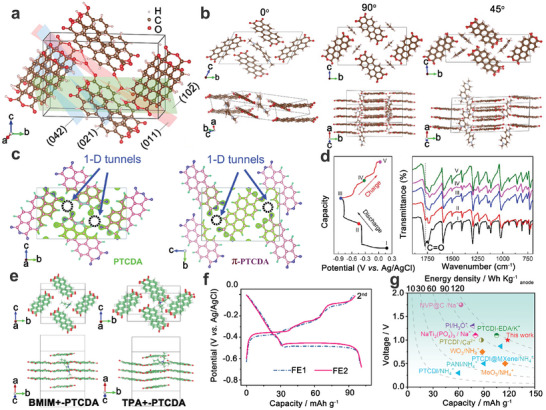
a) The simulation of the PTCDA unit cell with the insertion of H_3_O^+^. a) Reproduced with permission.^[^ ^108^ ^]^ Copyright 2017, Wiley‐VCH. b) Simulated MV^2+^‐inserted into the PTCDA unit cell with different angel (0°, 90° and 45°). b) Reproduced under the terms of the CC‐BY Creative Commons Attribution 4.0 International license (https://creativecommons.org/licenses/by/4.0).^[^ ^109^ ^]^ Copyright 2019, The Authors, published by Springer Nature. c) The 1D molecular tunnels (marked with circles) are walled by the terminal oxygen in PTCDA crystals. c) Reproduced with permission.^[^ ^110^ ^]^ Copyright 2020, the Royal Society of Chemistry. d) GCD curves of PTCDA at a current density of 0.1 A g^−1^ and the corresponding ex situ FT‐IR spectroscopy. d) Reproduced with permission.^[^ ^111^ ^]^ Copyright 2023, American Chemical Society. e) Simulated BMIM^+^‐inserted and TPA^+^‐inserted PTCDA, f) GCD curves of PTCDA at TPABr‐containing and BMIMBr‐containing aqueous electrolytes, g) Comparison of electrochemical performance of aqueous energy storage devices. e‐g) Reproduced with permission.^[^ ^112^ ^]^ Copyright 2024, Elsevier B.V.

The organic conjugated PTCDA molecule, a well‐known commercial red pigment, consists of two perylene cores and two anhydride groups, with the molecular formula C_24_H₈O₆. Notably, among all aromatic organic molecular crystals, PTCDA exhibits a long‐range ordered crystal structure and large interstitial sites within its unit cell due to the π–π stacking interactions of its planar molecules.^[^ ^105^ ^]^ This makes it an ideal model compound for hosting large‐sized redox charge carriers.^[^ ^36^ ^]^ For example, PTCDA was initially investigated as electrode materials for non‐aqueous Na^+^‐ion and K^+^‐ion batteries, showing good charge storage capability.^[^ ^106^, ^107^ ^]^ Later, Ji's group confirmed for the first time that the crystalline PTCDA showed reversible H_3_O^+^‐storage capability in a 1 M H_2_SO_4_ aqueous electrolyte, delivering a high capacity of 85 mAh g^−1^ at a current density of 1 A g^−1^.^[^ ^108^ ^]^ Furthermore, as shown in **Figure** **8**a, the H_3_O^+^ ions can be inserted into the large voids formed by the stacked PTCDA molecules in the unit cell. Additionally, in 2019, the same group demonstrated that a large charge carrier (methyl viologen ion, named MV^2+^) can be reversibly inserted into the interstitial sites between the columns of the stacked PTCDA molecules with a specific angle of 45° (Figure 8b),^[^ ^109^ ^]^ where a 0.1 M methyl viologen dichloride aqueous solution was used as the electrolyte. Subsequently, Lu's group further demonstrated that enhancing the π‐π stacking interaction between the molecular layers in the PTCDA crystal can promote electron transportation, and significantly improve the ion diffusion kinetics due to the activated 1D molecular tunnels (Figure 8c).^[^ ^110^ ^]^ Therefore, PTCDA organic molecular crystals with large open interstitial sites are expected to be promising host materials for NH_4_^+^‐ion storage. For example, Dong's group confirmed the NH_4_^+^‐ion storage mechanism within the PTCDA anode via ex situ XRD and FT‐IR characterizations in a 1 M (NH_4_)_2_SO_4_ electrolyte.^[^ ^111^ ^]^ Ex situ XRD spectra demonstrated that the lattice of the PTCDA anode underwent shrinkage and expansion during the charge/discharge process when NH_4_^+^ interacts with the PTCDA anode. Meanwhile, as shown in the ex situ FT‐IR spectra (Figure 8d), the intensity of the carbonyl groups (∼1773 cm^−1^) in PTCDA first weakened and then recovered during the discharge and charge process, indicating the reversible redox reactions between NH_4_^+^ and the PTCDA anode. Subsequently, the as‐assembled MnO_2_//PTCDA full cell delivered a discharged capacity of ∼100 mAh g^−1^ at a current density of 0.1 A g^−1^ based on the active mass loading of the cathode. Even at a power density of 8211.6 W kg^−1^, the MnO_2_//PTCDA full cell showed an energy density of 68.2 W h kg^−1^. However, the full cell suffered from severe capacity attenuation (51.8% of capacity retention after 4000 cycles), attributed to the dissolution of PTCDA during the redox process. Tang's group made significant progress by demonstrating that the addition of 1‐butyl‐3‐methylimidazolium bromide (BMIMBr) and tetrapropylammonium bromide (TPABr) into an aqueous ammonium bromide (NH₄Br) electrolyte (7 m NH₄Br + 1 m BMIMBr or 7 m NH₄Br + 1 m TPABr) improved the reversibility of NH₄⁺‐ion storage in the PTCDA anode.^[^ ^112^ ^]^ This enhancement is attributed to the insertion of organic cations (BMIM⁺ and TPA⁺) into the electrode. As shown in Figure 8e, the BMIM⁺ cation can fully intercalate between the molecular layers of the stacked PTCDA, while the TPA⁺ cation, with its four propyl chains, can only partially insert into the PTCDA matrix. Remarkably, the PTCDA anode exhibited a reversible capacity of ≈98 mAh g⁻¹ in the TPABr‐ and BMIMBr‐containing aqueous electrolytes, which is higher than the ≈80 mAh g⁻¹ capacity observed in the NH₄Br electrolyte (Figure 8f). The fully assembled aqueous battery delivered a discharge capacity of 118 mAh g⁻¹ at a current density of 0.5 A g⁻¹ and achieved an energy density of 113 Wh kg⁻¹, based on the active mass loading of the anode (Figure 8g).

**Figure 9:**
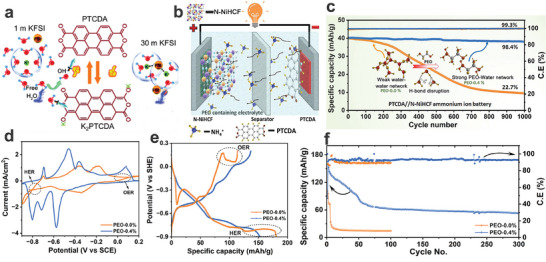
a) The energy storage mechanism of the PTCDA electrode in water‐in‐salt electrolytes. a) Reproduced with permission.^[^ ^88^ ^]^ Copyright 2020, the Royal Society of Chemistry. b) Illustration of the N‐NiHCF//PTCDA full cell, and c) cycling stability. d) CV curves, e) GCD curves, and f) cycling performance of the PTCDA anode in the pure aqueous electrolyte and PEO‐modified electrolyte. b–f) Reproduced with permission.^[^ ^118^ ^]^ Copyright 2023, American Chemical Society.

Actually, when PTCDA undergoes a reversible charge storage process in aqueous‐based electrolytes, it faces significant dissolution issues, leading to inferior cycling stability.^[^ ^113^ ^]^ Fortunately, the introduction of high concentration salt electrolytes, water‐in‐salt electrolytes (**Figure** **9**a), and hydrated molten inorganic salt electrolytes can effectively suppress the dissolution of PTCDA electrode materials, due to the reduced amount of “free” water.^[^ ^88^, ^114^, ^115^ ^]^ However, the use of high‐concentration salts inevitably increases costs and poses challenges such as higher viscosity and lower ion mobility. Introducing low‐cost additives (e.g., glucose) into aqueous electrolytes can modulate the solvation structure and suppress side reactions.^[^ ^116^ ^]^ Therefore, selecting appropriate electrolyte additives and understanding their impact on the fundamental structure of electrolytes are essential for enhancing the electrochemical performance of aqueous batteries.^[^ ^117^ ^]^ Similarly, Wang's group first reported that poly(ethylene oxide) (PEO) was used as an organic polymer‐based electrolyte additive to modify a 1 m ammonium acetate electrolyte.^[^ ^118^ ^]^ The results showed that the assembled ammoniated nickel Prussian blue (N‐NiHCF)//PTCDA full cell (Figure 9b) delivered high capacity retention of 98.4% after 1000 cycles in the PEO‐modified ammonium acetate electrolyte (Figure 9c). The excellent cycling stability of the full cell can be ascribed to the reconstruction of hydrogen‐bonding network structure between the PEO and water molecules, which can suppress side reactions. Besides, the hydrogen‐bonding interaction between NH_4_^+^ and PEO promotes ion migration along the PEO‐water network. Figure 9d,e shows the CV and GCD curves of the PTCDA anode in a 1 m NH_4_OAc electrolyte and a PEO‐modified 1 m NH_4_OAc electrolyte. Apparently, the PTCDA anode exhibited a stable electrochemical potential window in the PEO‐modified aqueous electrolyte, while side reactions (e.g., HER and OER) were observed in the pure aqueous electrolyte. Meanwhile, compared to the rapid capacity decay of the PTCDA anode in the pure aqueous electrolyte, the PTCDA anode demonstrated relatively stable cycling performance in the PEO‐modified electrolyte (Figure 9f), suggesting that the PEO additive can effectively improve the stability of the PTCDA anode. In the future, the exploration of novel electrolyte additives provides a feasible approach to achieving sustainable AAIBs with ultra‐stable cycling performance.

##### AAIBs Based on Other Small Organic Molecules

**Figure 10:**
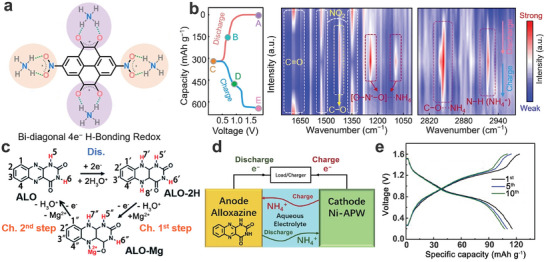
a) Illustration of lock‐and‐key hydrogen‐bonding networks between DNPT and NH_4_^+^ charge carrier. b) GCD curves of Zn//DNPT in an aqueous NH_4_OTF electrolyte and the corresponding ex situ FT‐IR spectra. a, b) Reproduced with permission.^[^ ^83^ ^]^ Copyright 2023, Wiley‐VCH. c) H‐storage chemistry in the ALO electrode. c) Reproduced with permission.^[^ ^120^ ^]^ Copyright 2020, the Royal Society of Chemistry. d) Illustration of the Ni‐APW//ALO AAIB, and e) the corresponding GCD curves at 4 C (1 C = 250 mA/g). d, e) Reproduced with permission.^[^ ^121^ ^]^ Copyright 2021, the Tsinghua University Press and Springer‐Verlag GmbH Germany, part of Springer Nature.

In addition to the typical aromatic organic crystals, it is essential to develop novel organic molecules as host materials for NH_4_^+^ storage. For example, Liu's group reported that 2,7‐dinitropyrene‐4,5,9,10‐tetraone (DNPT) interacts with NH_4_^+^ to form stable lock‐and‐key hydrogen‐bonding networks (**Figure** **10**a), which can not only significantly improve the stability of DNPT in aqueous electrolytes, but also can enhance the diffusion kinetics of NH_4_^+^ charge carrier.^[^ ^83^ ^]^ Ex situ FT‐IR spectra were performed to further demonstrate the structural evolution of the DNPT cathode with the interaction of NH_4_^+^ (Figure 10b). Obviously, in addition to the reversible transformations between the carbonyl groups (C═O, 1691 cm^−1^) and C─O bands (1421 cm^−1^), a new peak (2845 cm^−1^) is ascribed to the stretching mode of the electrostatic interaction (C─O^‐^···H_4_N^+^) during the NH_4_^+^‐ion storage process. Meanwhile, the nitro groups of DNPT can also reversibly store NH_4_^+^ charge carrier and form the NH_4_^+^‐interacted nitro species (H_4_N^+^···[^‐^O‐N‐O^‐^]···^+^NH_4_). Therefore, DNPT with carbonyl/nitro redox‐active centers possesses unique NH_4_^+^‐ion storage properties, holding great potential as electrode materials for high‐performance AAIBs. Additionally, Tao's group demonstrated that alloxazine (ALO) is a promising host material for H^+^ uptake/removal (Figure 10c), due to its fascinating structural characteristics and high theoretical specific capacity.^[^ ^119^, ^120^ ^]^ Subsequently, the same group further investigated the NH_4_^+^‐ion storage capability of the ALO anode in a 1 M (NH_4_)_2_SO_4_ aqueous electrolyte. The results demonstrated that the ALO electrode exhibited pseudocapacitive NH_4_^+^‐ion storage behavior and delivered a high discharge specific capacity of 138.6 mAh g^−1^ at a current density of 1 A g^−1^, along with excellent rate performance (a specific capacity of 120 mAh g^−1^ at a current density of 10 A g^−1^). The full battery was fabricated by using the ALO anode and Ni‐APW ((NH_4_)_2_Ni[Fe(CN)_6_]) cathode (Figure 10d), showing a specific capacity of 107 mAh g^−1^ at a current density of 1 A g^−1^ (Figure 10e) and a high energy density of 122.5 Wh kg^−1^. This work opens new avenues for the discovery of high‐capacity anode materials for AAIBs.

#### 2.4.2 Redox‐Active Organic Polymers

Polymeric materials are conspicuous because of their exceptional chemical stability, precisely designed structures, favorable electrical conductivity, and outstanding stretched and bending properties, in contrast to small organic molecules and metal oxides.^[^ ^122^ ^]^ Currently, polymeric materials including natural polymers (e.g., cellulose, sodium alginate, chitosan, etc.) and synthesized polymers (e.g., PANI, polydopamine, PPy, polyvinylidene fluoride), play a crucial role in the design and fabrication of advanced energy storage devices.^[^ ^123^ ^]^ For example, a variety of polymers have been reported as electrode materials, separators, protective coatings, hydrogel electrolytes, and electrolyte additives for the fabrication of high‐performance aqueous zinc‐ion batteries.^[^ ^124^ ^]^ Therefore, the precise molecular design of polymers and a clear understanding of the relationship between their structures and energy storage capabilities will advance the application of polymers in aqueous energy storage devices. Additionally, polymers possess structural flexibility, and large and adjustable internal voids, enabling them to effectively store large‐sized charge carriers and mitigate structural damage during charge carrier insertion.^[^ ^125^ ^]^ This renders polymers highly promising as host materials for accommodating NH_4_^+^.^[^ ^126^, ^127^ ^]^

##### Aromatic Carbonyl Compound‐Derived Polymers

**Figure 11:**
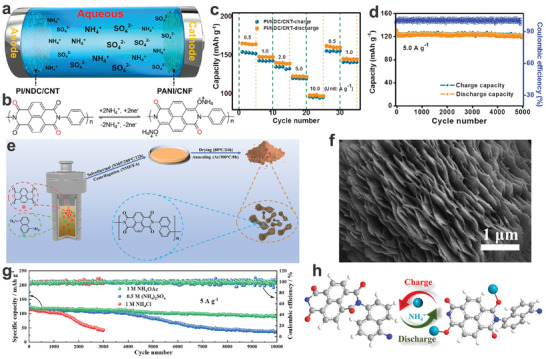
a) Illustration of all‐organic AAIBs. b) redox mechanisms, c) rate performance, and d) cycling stability of PI. a,c,d) Reproduced with permission.^[^ ^131^ ^]^ Copyright 2020, Elsevier B.V. e) Synthesis route of PNNI. f) SEM image, g) cycling performance in different electrolytes, and h) Possible energy storage mechanisms of PNNI. e‐h) Reproduced with permission.^[^ ^132^ ^]^ Copyright 2023, American Chemical Society.

Although aromatic compounds such as NTCDA, PTCDI, and PTCDA have proven to be effective as NH_4_^+^‐ion storage electrode materials, these small molecules suffer from inevitable dissolution during the charge/discharge process. However, polymers based on these aromatic compounds have been explored to mitigate the dissolution of electrode materials. For example, in 2019, Zhang's group used n‐type 1,4,5,8‐naphthalenetetracarboxylic dianhydride‐derived polyimide (PI) as the anode and p‐type poly(2,2,6,6‐tetramethylpiperidinyloxy‐4‐ylmethacrylate) (PTMA) as the cathode to assemble an aqueous ammonium dual‐ion battery in a 1 M (NH_4_)_2_SO_4_ aqueous electrolyte, where the n‐type PI and p‐type PTMA can store cations (NH_4_^+^) and anions (SO_4_^2‐^), respectively.^[^ ^128^ ^]^ Obviously, the PI anode showed a high specific capacity of 157.3 mAh g^−1^ at a current density of 0.5 A g^−1^. The as‐fabricated PI//PTMA full cell delivered an excellent energy density of 51.3 W h kg^−1^ and a high power density (15.8 kW kg^−1^). In addition, the PI//PTMA full cell exhibited stable cycling performance as well as high capacity retention of 86.4% after 10 000 cycles at a current density of 5 A g^−1^. Later, Ji's group investigated the K^+^/NH_4_^+^ insertion behavior of 1,4,5,8‐naphthalenetetracarboxylic dianhydride‐derived polyimide (PNTCDA) anode in a 25 m KOAc electrolyte and a 25 m NH_4_OAc electrolyte, respectively.^[^ ^129^ ^]^ The results show that the PNTCDA anode as NH_4_^+^‐ion storage electrode materials delivered a higher capacity (≈160 mAh g^−1^ at 0.16 A g^−1^) and relatively high capacity retention of 88.7% after 30 000 cycles. Until now, PNTCDA, as an excellent NH_4_^+^‐ion storage anode material, is still being used to match with inorganic electrode materials (e.g., (NH_4_)_2_V_6_O_16_∙1.5 H_2_O) for assembling AAIBs.^[^ ^130^ ^]^ In 2020, Liu's group synthesized a flexible and highly porous electroactive PI‐based nanofibrous membrane (named as PI/NDC/CNT) through an electrospinning process followed by pyrolysis and imidization treatments.^[^ ^131^ ^]^ The membrane is composed primarily of nitrogen‐doped carbon (NDC), carbon nanotubes (CNT), and the polycondensation product of NTCDA and *p*‐phenylenediamine. Then, the all‐organic AAIB was fabricated by employing PI/NDC/CNT as the anode and PANI/carbon nanofiber (PANI/CNF) as the cathode (**Figure** **11**a). As shown in Figure 11b, the C═O groups of PI can interact with NH_4_^+^ charge carriers to reversibly form or disassociate the ammonium enol compound during charge/discharge process. The results showed that the PI/NDC/CNT anode delivered a satisfactory rate capability (Figure 11c) and excellent cycling performance (87.9% capacity retention after 5000 cycles at a current density of 5 A g^−1^) (Figure 11d). Finally, the assembled PI/NDC/CNT//PANI/CNF full cell exhibited a high discharge capacity of 136.7 mAh g^−1^ and an impressive energy density of 114.3 W h kg^−1^, at a power density of 18.6 kW kg^−1^. Besides, Cao's group reported that a poly(1,4,5,8‐naphthalenetetracar‐boxylic anhydride naphthylamine) imine (PNNI) was prepared by the polycondensation of NTCDA and 1,5‐naphthalenediamine (1,5‐NDA) via a solvothermal reaction (Figure 11e).^[^ ^132^ ^]^ The obtained PNNI with carbonyl functional groups showed an ordered nanoflake‐like structure (Figure 11f), which promotes optimized electrolyte infiltration and improves the diffusion kinetics of NH_4_^+^. Furthermore, the electrochemical performance of the PNNI was tested in a 1 M NH_4_OAc electrolyte (neutral), a 0.5 M (NH_4_)_2_SO_4_ electrolyte (weakly acidic), and a 1 M NH_4_Cl (weakly acidic) electrolyte, to demonstrate the effect of H^+^ on the deintercalation of NH_4_^+^ within the PNNI electrode. In contrast, the PNNI electrode exhibited stable long‐term cycling performance in the 1 M NH_4_OAc aqueous electrolyte (Figure 11g), which may be attributed to the structural collapse of the PNNI electrode under a weakly acidic environment. Moreover, the NH_4_^+^‐ion storage mechanism in the PNNI electrode is displayed in Figure 11h. Finally, the full cell employing PNNI as the anode and Ni‐APW ((NH_4_)_2_Ni[Fe(CN)_6_]) as the cathode, delivered an energy density of 68.7 Wh Kg^−1^ at a power density of 383.8 W kg^−1^.

As discussed above, PTCDA and PTCDI molecules have been widely used as anodes in AAIBs. However, the inevitable dissolution of small molecules has driven researchers to focus on electrolyte engineering to improve the stability of electrode materials. Fortunately, some PTCDA‐derived polymers still use the carbonyl groups of PTCDA as the electrochemically active centers to accommodate charge carriers (e.g., Na^+^),^[^ ^133^ ^]^ and their structures can be tuned via introducing short alkyl chains or thioether bonds between the PTCDA monomers, showing enhanced electronic conductivity and improved stability.^[^ ^134^, ^135^ ^]^ Meanwhile, the PTCDI derivatives, after the polymerization of PTCDA with other small molecules (e.g., 2‐aminoanthraquinone), have been shown to significantly improve the electrochemical properties of electrode materials during the charge/discharge process.^[^ ^136^ ^]^ However, although the N,N'‐ditridecylperylene‐3,4,9,10‐tetracarboxylic diimide (PTCDI‐C_12_), urea‐perylene diimide polymer (UP) and hydrazine‐perylene diimide polymer (HP) were successfully synthesized and saved as the host materials for NH_4_^+^‐ion storage in a 1 M (NH_4_)_2_SO_4_ aqueous electrolyte.^[^ ^137^ ^]^ In the future, these ameliorative strategies for PTCDA‐derived polymers and PTCDI derivatives will contribute to the vigorous development of organic electrode materials in AAIBs. Additionally, exploring novel redox‐polymers to store NH_4_^+^ charge carriers is vital for high‐performance AAIBs. For example, Wang's group prepared an organic poly(1,5‐naphthalenediamine) (poly(1,5‐NAPD)), which mainly involves the reversible conversion of C═N/C─N‐ during the NH_4_^+^ insertion/extraction.^[^ ^138^ ^]^ The poly(1,5‐NAPD) showed fast kinetics in a concentrated NH_4_OAc electrolyte as well as a high discharge specific capacity of 141 mAh g^−1^ at a current density of 1 A g^−1^. The full AAIB was assembled by employing poly(1,5‐NAPD) as the anode and Prussian blue/CNT composites (NiHCF@CNTs) as the cathode, exhibiting a high energy density of 31.8 Wh kg^−1^ and operated across a wide temperature range (−40 to 80 °C). This work has sparked significant interest in the search for novel electrode materials for AAIBs.

##### Conventional Conductive Polymers

**Figure 12:**
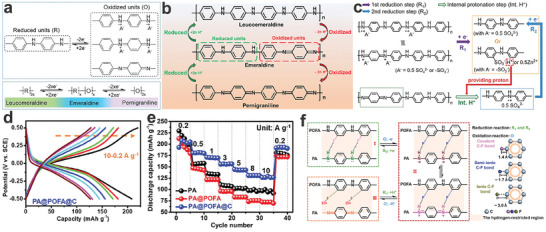
a) Three oxidation states of PANI. a) Reproduced with permission.^[^ ^146^ ^]^ Copyright 2021, Elsevier B.V. b) The structural changes of PANI. b) Reproduced with permission.^[^ ^149^ ^]^ Copyright 2024, Wiley‐VCH. c) Working mechanism of self‐doped PANI. c) Reproduced with permission.^[^ ^154^ ^]^ Copyright 2018, Wiley‐VCH. d) Charge‐discharge curves, e) rate performance, and f) the hydrogen‐restricted region in PA@POFA@C. d‐f) Reproduced with permission.^[^ ^149^ ^]^ Copyright 2024, Wiley‐VCH.

Conventional conductive polymers (CPs), such as PANI, polythiophene, PPy, polyindole and their derivates have gained wide attention in the field of energy storage devices, due to their low cost, facile synthesis, excellent mechanical flexibility and inherently high conductivity within the polymer chains.^[^ ^139^, ^140^, ^141^, ^142^ ^]^ As the most typical representative of CPs, PANI exhibits apparent merits of low‐cost monomer, high electrochemical activity, and unique doping/dedoping chemistry (protonic acid doping and oxidative doping).^[^ ^143^, ^144^, ^145^ ^]^ Moreover, as shown in **Figure** **12**a, PANI with long‐range conjugated structures shows three oxidation states, such as fully reduced (leucomeraldine), half oxidized (emeraldine) and fully oxidized (pernigraniline) states.^[^ ^146^, ^147^, ^148^ ^]^ The different states of PANI can be reversibly converted via oxidation or reduction process (Figure 12b).^[^ ^149^, ^150^ ^]^ However, upon deprotonation, PANI becomes fully oxidized to pernigraniline, which is prone to hydrolysis in aqueous solutions, forming soluble oligoaniline. This leads to structural collapse, degradation of PANI, and a significant reduction in capacity.^[^ ^151^, ^152^ ^]^ Actually, the electrochemical characterization of PANI can be dated back to 1980 using a 0.1 M H_2_SO_4_ solution as the electrolyte.^[^ ^153^ ^]^ Therefore, it is necessary to maintain a high proton concentration in aqueous electrolytes to ensure the normal operation of PANI. However, a highly acidic environment will inevitably lead to the corrosion of stainless steel current collectors and compromise the stability of electrode materials. Sun's group prepared a stable sulfo‐self‐doped PANI (PANI‐S) electrode by introducing ‐SO_3_^‐^ group into the PANI molecular chains, acting as a proton reservoir.^[^ ^154^ ^]^ The ‐SO_3_^‐^ self‐dopant promotes a high proton concentration around the PANI chains (Figure 12c), leading to excellent electrochemical activity of PANI in a 1 M ZnSO_4_ electrolyte (weakly acidic). This work further provides a promising direction for the application of PANI in the field of energy storage.

Since then, PANI has been widely used for the storage of various charge carriers (e.g., H^+^, Na^+^, and Zn^2+^) as well as for the protection of Zn anodes.^[^ ^155^, ^156^, ^157^, ^158^ ^]^ Currently, with the emergence of AAIBs, PANI has also been employed as host materials for NH_4_^+^‐ion storage. For example, in 2020, Wang's group demonstrated for the first time that doping PANI‐based polymers can efficiently store NH_4_^+^ ions.^[^ ^159^ ^]^ Detailedly, the electrochemical performance of emeraldine salt state PANI (ES‐PANI) and emeraldine base state PANI (EB‐PANI) was tested in a 0.5 M (NH_4_)_2_SO_4_ aqueous electrolyte to reveal the mechanism of NH_4_^+^‐ion storage. The Cl^‐^ ‐dopant endows the ES‐PANI with a higher conductivity. In addition, the self‐doping of ES‐PANI enables the reversible transformation between quinoid and benzenoid rings due to the NH_4_^+^ uptake/removal. Compared to EB‐PANI, the self‐doped ES‐PANI with anions delivered a higher discharge capacity of 160 mAh g^−1^ at a current density of 1 A g^−1^. Ex situ XPS results revealed the formation of ‐NH‐ and ‐NH^+^‐ groups during the reduction process, confirming the interconversion of quinoid and benzenoid rings. This work has aroused research interest in PANI‐based derivatives as electrode materials for high‐performance AAIBs. Later, the same group constructed a full NH_4_^+^‐ion cell in an aqueous 1 M (NH_4_)_2_SO_4_ electrolyte, employing NH_4_V_3_O_8_·2.9H_2_O nanobelts as the cathode, PANI as the anode, and the full cell showed a specific capacity of 121 mAh g^−1^ at a current density of 0.1 A g^−1^.^[^ ^126^ ^]^ Besides, the flexible NH_4_V_3_O_8_·2.9H_2_O//PANI full cell in a concentrated hydrogel electrolyte (PAM/NH_4_Cl hydrogel) demonstrated excellent mechanical robustness and flexibility. Similarly, Wang's group prepared PANI nanorods grown on carbon fiber (CF@PANI) via electropolymerization, delivering a discharge capacity of 77 mAh g^−1^ at a current density of 0.1 A g^−1^ in a 1 M (NH_4_)_2_SO_4_ aqueous electrolyte.^[^ ^160^ ^]^ Subsequently, the NH_4_^+^‐ion full cell was fabricated by using CF@PANI as the anode and urchin‐like NH_4_V_4_O_10_ coated on carbon fiber (CF@NH_4_V_4_O_10_) as the cathode, exhibiting a high discharge capacity of 167 mAh g^−1^ at a current density of 0.1 A g^−1^, based on the total active mass loading of both the anode and cathode. Finally, the fiber‐shaped NH_4_^+^‐ion full cell was assembled inside a heat‐shrinkable tube, showing good flexibility and excellent electrochemical performance. Therefore, PANI has also emerged as a suitable and efficient anode material for AAIBs. For example, a PANI anode matched with a cubic copper hexacyanoferrate (CuHCF) cathode was used to fabricate a full NH_4_^+^‐ion cell, delivering a discharge capacity of 55.3 mAh g^−1^ at a current density of 2 A g^−1^ and high capacity retention of 74.3% after 1240 cycles.^[^ ^161^ ^]^

In order to further improve the electrochemical performance of PANI, Sun's group prepared polyaniline@poly(o‐fluoroaniline)@carbon layer (PA@POFA@C) composites by electrodeposition followed by a hydrothermal method.^[^ ^149^ ^]^ The composites exhibited a stable structure for NH_4_^+^‐ion storage, delivering a high discharge capacity of 208 mAh g^−1^ at a current density of 0.2 A g^−1^ in a 1 M NH_4_OAc electrolyte (pH ≈ 7.2) (Figure 12d,e). As displayed in Figure 12f, the movement of protons can be restricted between the PA and POFA layer (forming a hydrogen‐restricted region), due to the confinement effect provided by the elastic C‐F bonds, which can be conducive to facilitating the complete reduction of pernigraniline and minimizes the undesirable transformations. Finally, the ammonium vanadate (NH_4_V_4_O_10_)//PA@POFA@C full cell was assembled by employing PA@POFA@C as the cathode, delivering a discharge capacity of 38 mAh g^−1^ at a current density of 0.2 A g^−1^ and an energy density of 13.89 Wh kg^−1^ at a power density of 72.87 W kg^−1^. Additionally, Sun's group used the sulfonic acid group (‐SO_3_^‐^H^+^) of 1,5‐Naphthalenedisulfonic acid (1,5‐NDSA) to restrain the escape of H^+^ and the group acted as a H^+^ reservoir, thus ensuring the reversible transformation between ‐NH‐ and –N═ in PANI.^[^ ^162^ ^]^ The PANI and 1,5‐NDSA hybrid material (PANI‐H^+^) exhibited a discharge capacity of 299.3 mAh g^−1^ at a current density of 1 A g^−1^ and maintained excellent capacity retention of ≈100% after 1000 cycles (following rate performance testing) at a current density of 10 A g^−1^ in a 1 M (NH_4_)_2_SO_4_ electrolyte mixed with NH_4_I and iodine (I_2_). Finally, the assembled PTCDA//PANI‐H^+^ ammonium‐iodine full cell delivered a good capacity retention rate of 87% after 1400 cycles. Interestingly, Zhang's group fabricated a flexible soft pack battery in a poly(vinyl alcohol)/NH_4_Cl (PVA/NH_4_Cl) hydrogel electrolyte, where PANI was used as the cathode to match with the VO_2_·xH_2_O (with rich oxygen defects) anode.^[^ ^163^ ^]^ The full battery exhibited outstanding flexibility and a high reversible capacity of 100 mAh g^−1^ at a current density of 1 A g^−1^. In addition to PANI, Wang's group reported that PPy is also an excellent host material for NH_4_^+^‐ion storage, which delivered a high discharge capacity of 125.77 mAh g^−1^ at a current density of 1 A g^−1^ and maintained capacity retention of 73.77% after 100 cycles in a 25 m NH_4_OAc electrolyte.^[^ ^164^ ^]^ Interestingly, the metal‐free all‐organic NH_4_^+^‐ion full cell was designed in a 19 m NH_4_OAc electrolyte by using PPy as the cathode and PANI as the anode, and its electrochemical performance was tested at 25 and 0 °C. The PPy//PANI full cell delivered a high capacity of 78.405 mA h g^−1^ at 25 °C and 49.083 mAh g^−1^ at 0 °C, both at a current density of 0.1 A g^−1^. This metal‐free design opens new avenues for the development of safer and more sustainable aqueous batteries.

#### 2.4.3 COFs

**Figure 13:**
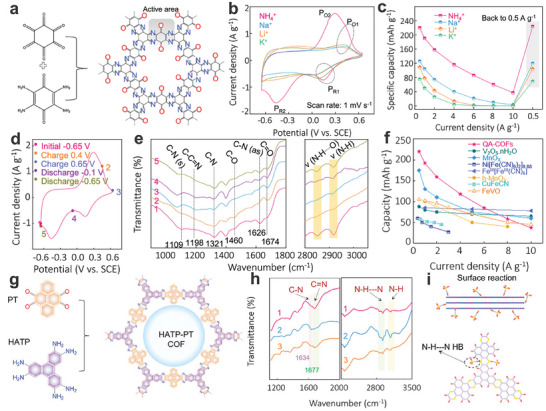
a) Synthesis and structure of the QA‐COF. b) CV curves, and c) rate capability of the QA‐COF in different cation‐containing electrolytes. d) CV curve, and e) corresponding ex situ FTIR spectra in a slightly acidic (NH_4_)_2_SO_4_ electrolyte (pH 5.88). f) NH_4_^+^‐ion storage capacity of the QA‐COF. a–f) Reproduced with permission.^[^ ^187^ ^]^ Copyright 2021, American Chemical Society. g) Preparation of the HATP‐PT COF. h) Ex situ FTIR spectra of the HATP‐PT COF. i) The interaction between the HATP‐PT COF and NH_4_^+^ ions. g‐i) Reproduced with permission.^[^ ^84^ ^]^ Copyright 2024, Wiley‐VCH GmbH.

Electrochemically active COFs, known for their crystalline long‐range order and porous polymeric nature, have garnered significant attention across various fields, including gas storage and separation, water treatment, sensing, and energy storage.^[^ ^165^ ^]^ Their appeal lies in intrinsic advantages such as customizable molecular structures, adjustable pore sizes, and excellent chemical stability.^[^ ^166^, ^167^ ^]^ Fundamentally, the skeleton of COFs is constructed by covalent bonds (e.g., boroxine, imines, imides, and azines)^[^ ^168^ ^]^ that connect repeated organic building blocks to form insoluble 2D^[^ ^169^ ^]^ and 3D^[^ ^170^ ^]^ polymeric frameworks. The large number of periodic organic units within the backbones of COFs contributes to the high porosity, ordered pore channels, and large surface area. Meanwhile, the structure of COFs can be accurately predesigned via integrating active groups (e.g., C═O, N═N, and C═N) and various redox‐active sites into their skeletons.^[^ ^171^ ^]^ Therefore, the selection of appropriate building blocks, active centers and stable linkages is crucial to optimize the electrochemical activities of COFs, such as redox‐active donor–acceptor (D–A) type COFs with bipolar carrier properties.^[^ ^172^ ^]^ Incipiently, Yaghi's group first successfully synthesized crystalline COFs (COF‐1, COF‐5) and 3D COFs.^[^ ^173^, ^174^ ^]^ Until now, various COFs have been served as electrode materials for rechargeable LIBs,^[^ ^175^, ^176^ ^]^ supercapacitors,^[^ ^177^, ^178^, ^179^ ^]^ aqueous proton batteries,^[^ ^180^ ^]^ aqueous acid/alkali batteries,^[^ ^23^, ^181^ ^]^ aqueous zinc batteries,^[^ ^182^, ^183^ ^]^ aqueous magnesium ion supercapattery,^[^ ^184^ ^]^ aluminum batteries,^[^ ^185^ ^]^ and aqueous calcium‐ion batteries.^[^ ^186^ ^]^ In 2021, Alshareef's group first revealed the intercalation mechanism of NH_4_^+^‐ions in a COF.^[^ ^187^ ^]^ The COF was synthesized using 2,3,5,6‐tetraminocyclohexa‐2,5‐diene‐1,4 dione and hexaketocyclohexane octahydrate via a solvothermal method (**Figure** **13**a), followed by activation at 200 °C before electrochemical tests (named as QA‐COF). The redox‐active area is the repeated unit containing carbonyl oxygen and pyrazine nitrogen inside the QA‐COF skeleton. Subsequently, the electrochemical performance of QA‐COF was tested in different electrolytes including (NH_4_)_2_SO_4_, Li_2_SO_4_, Na_2_SO_4_, and K_2_SO_4_ (0.5 m each). In contrast, QA‐COF as the NH_4_^+^‐ion storage material, exhibited a larger integrated area (Figure 13b), delivering a high discharge capacity of 220.4 mAh g^−1^ at a current density of 0.5 A g^−1^ (Figure 13c), along with excellent cycling stability. The high electrochemical performance can be attributed to the H‐bond‐induced interaction with NH_4_^+^ (N‐H···O and N‐H···N HBs) and the unique solvation behavior of NH_4_^+^ ions. H‐bond interaction mechanism can be confirmed by ex situ FTIR at different potentials (Figure 13d). As shown in Figure 13e, the vibration band of C═O (1674 cm^−1^) first strengthened and then weakened during the redox process. Besides, the vibration intensity of the N‐H···O and N‐H···N HBs (2850 cm^−1^) reversibly decreased and increased during the charge/discharge process. Finally, the QA‐COF still delivered a higher NH_4_^+^‐ion storage capacity compared to other inorganic materials, as shown in Figure 13f. This work is of great significance for the application of COFs in high‐performance AAIBs. In the future, novel COFs should be developed as NH_4_^+^‐ion storage materials. Recently, the same group successfully designed an aza‐based COF (HATP‐PT COF) (Figure 13g) with a high conjugation degree and good crystalized porous structures, which was used as the anode in rocking‐chair AAIBs.^[^ ^84^ ^]^ In addition, sucrose was introduced as an electrolyte additive (2 m NH_4_OTf@sucrose as the electrolyte) to further optimize the electrochemical performance of the HATP‐PT COF anode and mitigate electrolyte side reactions. The HATP‐PT COF showed a relatively negative voltage window (−1–0.3 V versus SCE) and good electrochemical reversibility in a 2 m NH_4_OTf@sucrose electrolyte. Meanwhile, the COF delivered a capacity of 108.5 mAh g^−1^ at a current density of 0.2 A g^−1^ and demonstrated exceptional cycling stability, without any capacity deterioration even after 20 000 cycles at a current density of 1 A g^−1^. Ex situ FT‐IR measurement of the HATP‐PT COF demonstrated that the C = N bonds were the main redox center for the storage of NH_4_^+^ (Figure 13h) and the N‐H···N HBs can be formed between the N atoms and NH_4_^+^ ions (Figure 13i). Finally, the full NH_4_^+^‐ions cell was fabricated using HATP‐PT COF as the anode, Prussian blue (CuHCF) as the cathode, and a 2 m NH_4_OTf@S as the electrolyte. The full cell exhibited a high discharge capacity of 30.4 mAh g^−1^ at a current density of 0.4 A g^−1^ (based on the total mass loading of both the anode and cathode) and maintained good capacity retention of 89% after 20 000 cycles. Interestingly, the successfully assembled NH_4_^+^ pouch full cell can power a LED diode and showed a wide temperature operating range (a specific capacity of 1 mAh at ‐20 °C), indicating great potential for practical applications.

#### 2.4.4 Organic/Inorganic Hybrid Materials

As mentioned above, strategies including polymerization, predesigned molecular structure, substitution of functional groups, and electrolyte optimization, can effectively suppress the dissolution of organic materials and improve their stability during the charge/discharge process. single‐component materials still fall short of meeting the growing demand for high‐capacity and long‐lasting electrodes in AAIBs. In particular, organic small molecules and many electronically insulating polymers exhibit poor electrical conductivity, leading to inefficient utilization of active materials and suboptimal rate capability.^[^ ^188^ ^]^ Therefore, organic compounds combined with a conductive substrate (e.g., graphene, MXene, CNT, etc.)^[^ ^189^, ^190^, ^191^ ^]^ to form organic/inorganic hybrid materials. These hybrid materials blend the favorable processability of organic compounds with the excellent electron transport capabilities of conductive inorganic components, providing a synergistic advantage for enhancing the performance of AAIBs. In addition to the materials mentioned, black phosphorus is a promising alternative as a conductive filler, owing to its excellent electrical conductivity and unique 2D structure.^[^ ^192^, ^193^, ^194^ ^]^ These properties can significantly enhance the overall performance of organic electrode materials in AAIBs.

##### Organic Compounds/Conductive Substrate Composites

Conductive substrates including carbon‐baed materials, graphene, MXene, and their derivatives, play a very important role in the electrochemical properties of organic materials, especially for small molecules. In recent years, various organic molecules have been coupled with high‐surface‐area conductive substrates via covalent/noncovalent interactions, enabling devices with enhanced capacity and energy density.^[^ ^195^, ^196^ ^]^ For example, by varying reduced graphene oxide (rGO) contents, Lin's group investigated the NH_4_^+^‐ion storage behavior of PTCDI‐rGO composites.^[^ ^197^ ^]^ The rGO nanosheets can improve the conductivity of the composites and faciliate the homogenous distribution of PTCDI, which can significantly accelerate the NH_4_^+^‐ion intercalation kinetics. The optimized PTCDI‐rGO electrode delivered a high capacity of 165 mAh g^−1^ at a current density of 0.5 A g^−1^ in a 32 m NH_4_OAc high‐concentration electrolyte. The PTCDI‐rGO (anode)//AC(cathode) full cell in a 32 m NH_4_OAc electrolyte exhibited a specific capacity of 15.3 mAh g^−1^ at a current density of 1 A g^−1^ and maintained a high capacity retention of 74% after 3000 cycles, along with a wide operating temperature range (−20–50 °C). In addition, redox‐active 7,7,8,8‐tetracyanoquinodimethane (TCNQ) molecules were uniformly dispersed on the surface of rGO foam to enhance the transportation of NH_4_^+^ and electrons.^[^ ^198^ ^]^ Compared to the pure TCNQ electrode, the TCNQ‐rGO composite showed an improved capacity of 92.7 mAh g^−1^ at a current density of 1 A g^−1^ and demonstrated long‐term cycling stability (capacity retention of 73.2% after 5000 cycles). However, such a high content of low‐activity conductive additives inevitably reduces both the weight and volumetric energy density of the device.

Different from carbon‐based conductive substrates, MXenes as novel 2D lamellar structure materials, possess many unique and excellent characteristics including metallic‐like conductivity, controllable interlayer space, abundant terminal groups, and good hydrophilicity, showing a high surface electrochemical activity.^[^ ^199^ ^]^ Especially, the abundant open 2D channels in the layered stacking of MXenes are expected to accommodate large charge carriers.^[^ ^200^ ^]^ As a typical example, titanium carbide (Ti_3_C_2_T_x_) MXenes show a high electrical conductivity (up to 15 100 S cm^−1^),^[^ ^201^ ^]^ abundant functional groups (e.g., ‐F, ‐O, ‐OH), as well as an intercalation pseudocapacitance mechanism.^[^ ^202^ ^]^ These properties enable them to function not only as electrode materials for storing charge carriers but also as conductive substrates supporting active materials.^[^ ^203^, ^204^ ^]^ Furthermore, the electrostatic interactions between organic compounds and MXene facilitate the construction of stable composite materials.^[^ ^205^ ^]^ Additionally, the functional groups on MXene can promote the polymerization of organic compounds on its surface, allowing for the rational design of stable and uniform organic/inorganic hybrid materials.^[^ ^206^ ^]^ Inspired by the aforementioned consideration, Gao's group prepared a free‐standing PTCDA/Ti_3_C_2_T_x_ MXene film via simple solution mixing and extraction filtration to investigate its NH_4_^+^‐ion storage capability.^[^ ^207^ ^]^ Then, the aqueous micro batteries were assembled using PTCDA/Ti_3_C_2_T_x_ MXene film as the anode and MnO_2_/CNTs as the cathode. The CV curve of the PTCDA/Ti_3_C_2_T_x_ MXene electrode showed the largest integral area. The corresponding GCD curve demonstrated that the PTCDA/Ti_3_C_2_T_x_ MXene delivered a high discharge capacity of 202.79 mAh g^−1^ at a current density of 0.5 A g^−1^. Importantly, the introduction of Ti_3_C_2_T_x_ MXene can significantly improve the stability of PTCDA, and the PTCDA/Ti_3_C_2_T_x_ MXene electrode delivered high capacity retention of 74.31% after 10 000 cycles. Interestingly, in order to further explore the NH_4_^+^‐ion storage capability of novel MXenes, Sun's group investigated the electrochemical behavior of V_2_CT_x_ MXene in aqueous (NH_4_)_2_SO_4_, NH_4_Cl, (NH_4_)_2_C_2_O_4_, NH_4_Me and NH_4_OAc electrolytes (0.5 M each).^[^ ^208^ ^]^ The results showed that V_2_CT_x_ MXene demonstrated pseudocapacitive behavior for NH_4_^+^‐ion storage, delivering a high specific capacity of 115.9 mAh g^−1^ at a current density of 1 A g^−1^ in a 0.5 M NH_4_OAc electrolyte. This work provides a new opportunity to design MXene‐based organic/inorganic hybrid materials for high‐performance AAIBs.

##### Organic Compounds/Metal Compounds Composites

Metal compounds (e.g., vanadium‐based oxide, manganese‐based oxide, molybdenum‐based compounds, PBAs, and their derivatives) with open frameworks and layered structures can provide large ionic channels to accommodate NH_4_^+^‐ions.^[^ ^209^ ^]^ Additionally, as host materials for NH_4_^+^‐ion storage, inorganic materials (e.g., metal oxides, sulfides) not only provide high capacity but also contribute to a higher operating working voltage compared to organic materials.^[^ ^210^, ^211^ ^]^ However, the further application of metal compounds in AAIBs has been limited, due to their intrinsically poor electrical conductivity, sluggish ion‐transport kinetics, underutilized active sites, and structural collapse caused by phase change.^[^ ^212^ ^]^ Fortunately, CPs can not only be polymerized in situ on the surfaces of metal compounds, but also can intercalate into the interlayer space of metal compounds, thereby improving the kinetics of ion insertion/extraction and enhancing the stability of composites.^[^ ^213^, ^214^ ^]^ The strong interaction between metal compounds and CPs effectively enhances the NH_4_^+^‐ion storage electrochemical performance of organic/inorganic hybrid materials.

Organic/inorganic composite materials have also been utilized in AAIBs. For example, Dai's group prepared MoS_2_@PANI composites as promising NH_4_^+^‐ion host materials, where PANI shell can effectively protect the nanorod structure of MoS_2_ and improve the hydrophilicity of the composite materials.^[^ ^215^ ^]^ Therefore, the MoS_2_@PANI electrode exhibited enhanced electron and ions transport dynamics in a 1 M NH_4_Cl electrolyte, showing a specific capacitance of 452 F g^−1^ at a current density of 1 A g^−1^. The assembled symmetric MoS_2_@PANI//MoS_2_@PANI device displayed an energy density of 59.8 Wh kg^−1^ at a power density of 725 W kg^−1^. Besides, the content of PANI can significantly affect the electrochemical properties of the composite materials. Yang's group prepared a PANI/PBA (Na_0.73_Ni[Fe(CN)_6_]_0.88_) composite with different content of PANI via in situ polymerization of aniline.^[^ ^216^ ^]^ The collaborative contribution of PANI and PBAs provides comprehensive performance improvements for NH_4_^+^‐ion storage. The optimized PANI/Na_0.73_Ni[Fe(CN)_6_]_0.88_ hybrid material delivered a discharge capacity of 92.5 mAh g^−1^ at a current density of 0.1 A g^−1^. Meanwhile, the full NH_4_^+^‐ion cell was fabricated by combining the PANI/PBAs cathode with the polyimide@MXene (PI@MXene) anode, showing a specific capacity of 52.5 mAh g^−1^ at a current density of 1 A g^−1^. In addition to PANI, Liu's group designed core‐shell structured vanadium oxide/polypyrrole composite materials (VO_x_@PPy) for AAIBs.^[^ ^33^ ^]^ The strong interaction between PPy and VO_x_ endowed the organic/inorganic hybrid material with a high specific capacity of 195.36 mAh g^−1^ at a current density of 0.2 A g^−1^ in a 0.5 M NH_4_OAc aqueous electrolyte. The aqueous NH_4_^+^‐ion full cell employing VO_x_@PPy as the anode and activated Ni‐Co double‐layered hydroxide material (Ni‐Co LDH‐A) as the cathode, showed a high energy density of 74.1 Wh kg^−1^ at a power density of 75.6 W kg^−1^. This work highlights the important role of organic/inorganic composites for excellent electrochemical performance of AAIBs.

The organic materials pre‐intercalation engineering strategy can also be employed for AAIBs. In addition to the in situ polymerization of organic materials on the surface of inorganic materials, conducting polymers can be intercalated into the layers of vanadium oxide to adjust the lattice spacing. This approach stabilizes the layered structure and enhances the mobility of charge carriers (e.g., Zn^2^⁺ or NH₄⁺).^[^ ^47^, ^217^ ^]^ Typically, PANI‐intercalated V_2_O_5_ with enlarged interlayer spacing can effectively tune the kinetics of NH_4_^+^‐ion insertion/extraction and significantly improve the electrochemical performance of host materials for NH_4_^+^‐ion storage, delivering a high capacity of 192.5 mAh g^−1^ at a current density of 1 A g^−1^.^[^ ^218^ ^]^ Besides, PANI and guest ions co‐inserted into host materials enhance both electron and ion transfer kinetics.^[^ ^219^ ^]^ Therefore, Zhang's group prepared K^+^/PANI co‐intercalated vanadium oxide hydration (KVO/PANI), where the synergistic effect of K^+^ and PANI can optimize the NH_4_^+^‐ion intercalation pseudocapacitive behavior.^[^ ^220^ ^]^ The KVO/PANI electrode exhibited a high capacity of 340 F g^−1^ at a current density of 0.5 A g^−1^. The quasi‐solid‐state device was assembled using KVO/PANI as the cathode, activated carbon (AC) as the anode, and PVA/NH_4_Cl gel as the electrolyte, delivering an energy density of 31.8 Wh kg^−1^. Additionally, as a guest molecule, poly(3,4‐ethylenedioxithiophene) (PEDOT) can also be intercalated into host materials to improve the electrochemical performance for NH_4_^+^‐ion storage.^[^ ^221^ ^]^ For example, Zhang's group designed a PEDOT‐intercalated vanadium oxide hydrate (VOH) (named as VOH/PEDOT), exhibiting a much higher capacity in a 1 M PVA/NH_4_Cl electrolyte (327 F g^−1^ at 0.5 A g^−1^) compared to a 1 M NH_4_Cl electrolyte (124 F g^−1^ at a current density of 0.5 A g^−1^).^[^ ^222^ ^]^ These intriguing results highlight the potential for high‐performance wearable AAIBs.

**Tables** **1** and **2** present a detailed comparison of the electrochemical performance of various organic electrode materials, evaluated in both three‐electrode and two‐electrode configurations. These comparisons offer a comprehensive overview of how organic materials perform across different system setups, providing valuable insights into their effectiveness and potential for application in NH_4_^+^‐ion storage. At present, the structure‐property relationships between molecular‐scale hydrogen bonding interactions and the macroscopic electrochemical performance of AAIBs, remain inadequately understood.

**Table 1:** The comparison of various organic electrodes in half cells.

Organic electrodes	Reference electrode	Counter electrode	Electrolyte	Out voltage	Capacity	Cycle life (retention)	Refs.
PTCDI	Ag/AgCl (Replaced KCl with NH_4_Cl)	Activated carbon	1 M (NH_4_)_2_SO_4_	−1.05−0.2 V	158.9 mAh g^−1^ at 0.24 A g^−1^	400 (≈90%)	[35]
PTCDI	Ag/AgCl (Saturated KCl)	Pt	Saturated (NH_4_)_2_SO_4_	−0.7−0.7 V	61.6 mAh g^−1^ at 1 C	250 (‐)	[50]
PTCDI	Ag/AgCl (Saturated KCl)	Pt‐coated Ti (Pt/Ti)	5.8 m (NH_4_)_2_SO_4_	−1−0.3 V	≈100 mAh g^−1^ at 5 C	5000 (≈98%)	[92]
PTCDI	Ag/AgCl	Activated carbon	21 m NH_4_TFSI	−1.1−0.2 V	≈140 mAh g^−1^ at 1 A g^−1^	300 (‐)	[95]
PTCDI	SCE	Graphite rod	2 m NH_4_OTf@ sucrose	−1.1−0.3 V	145 mAh g^−1^ at 0.1 A g^−1^	500 (82.7%)	[100]
PTCDI	SCE	–	1 M NH_4_OAc with EG	−1−0 V	77.9 mAh g^−1^ at 1 A g^−1^	1000 (72.4%)	[101]
PTCDI	Ag/AgCl	AC pellets	1 m NH_4_OAc with EG	−1−0.3 V	≈130 mAh g^−1^ at 0.12 A g^−1^	5000 (64%)	[60]
PTCDI	Organic Ag/Ag^+^	Carbon cloth	1 m NH_4_PF_6_/ ADN‐EMC	−1‐0.4 V	≈70 mAh g^−1^ at 0.08 A g^−1^	40 (‐)	[70]
PTCDA	Ag/AgCl (1 M KCl)	Activated carbon films	1 M (NH_4_)_2_SO_4_	−0.9−0.15 V	≈180 mAh g^−1^ at 0.1 A g^−1^	1500 (≈72.3%)	[111]
PTCDA	Ag/AgCl (Saturated)	Platinum wire	7 m NH_4_Br + 1 m TPABr	−0.7−0 V	≈98 mAh g^−1^ at 0.5 A g^−1^	–	[112]
PTCDA	SCE	Carbon rod	1 m NH_4_OAc with 0.4% PEO	−0.9−0.2 V	151.02 mAh g^−1^ at 0.1 A g^−1^	300 (‐)	[118]
Alloxazine (ALO)	Ag/AgCl	Platinum plate	1 M (NH_4_)_2_SO_4_	−0.8−0.1 V	138.6 mAh g^−1^ at 1 A g^−1^	1500 (80%)	[121]
PI	SCE	Active carbon film	1 M (NH_4_)_2_SO_4_	−0.9−0.1 V	157.3 mAh g^−1^ at 0.5 A g^−1^	–	[128]
PNTCDA	Ag/AgCl (Saturated KCl)	Activated carbon	25 m NH_4_OAc	−1−0 V	≈160 mAh g^−1^ at 0.16 A g^−1^	30 000 (88.7%)	[129]
PI/NDC/CNT	SCE	Carbon cloth	1 M (NH_4_)_2_SO_4_	−0.9−0.1 V	161 mAh g^−1^ at 0.5 A g^−1^	5000 (87.9%)	[131]
PNNI	SCE	Carbon rod	1 M NH_4_OAc	−0.9−0.2 V	147.7 mAh g^−1^ at 0.1 A g^−1^	10 000 (80.2%)	[132]
poly(1,5‐NAPD)	Ag/AgCl	Activated carbon	19 m NH_4_OAc	−0.8−0.4 V	141 mAh g^−1^ at 1 A g^−1^	1000 (94%)	[138]
ES‐PANI	Ag/AgCl (1 M KCl)	Graphite rod	0.5 M (NH_4_)_2_SO_4_	−0.2−0.8 V	160 mAh g^−1^ at 1 A g^−1^	100 (82%)	[159]
CF@PANI	Ag/AgCl (Replaced KCl with NH_4_Cl)	Graphite rod	1 M (NH_4_)_2_SO_4_	−0.1−0.5 V	77 mAh g^−1^ at 0.1 A g^−1^	100 (81.8%)	[160]
PA@POFA@C	SCE	Platinum plate	1 M NH_4_OAc	−0.5−0.5 V	208 mAh g^−1^ at 0.2 A g^−1^	2000 (88.24%)	[149]
PPy	Ag/AgCl (Saturated)	Graphite rod	25 m NH_4_OAc	−1−0.5 V	125.77 mAh g^−1^ at 1 A g^−1^	100 (73.77%)	[164]
QA‐COF	SCE	–	0.5 m (NH_4_)_2_SO_4_	−0.65−0.65 V	220.4 mAh g^−1^ at 0.5 A g^−1^	7000 (‐)	[187]
HATP‐PT COF	SCE	Active carbon	2 m NH_4_OTf@ sucrose	−1−0.3 V	108.5 mAh g^−1^ at 0.2 A g^−1^	20 000 (no attenuation)	[84]
PTCDI‐rGO	Ag/AgCl (Saturated KCl)	Carbon rod	32 m NH_4_OAc	−1.1−0.3 V	165 mAh g^−1^ at 0.5 A g^−1^	100 (93%)	[197]
TCNQ‐rGO	Ag/AgCl	Carbon rod	1 M (NH_4_)_2_SO_4_	−0.35−0.8 V	92.7 mAh g^−1^ at 1 A g^−1^	5000 (73.2%)	[198]
PTCDA/Ti_3_C_2_T_x_ MXene	Ag/AgCl	–	(NH_4_)_2_SO_4_‐PAM hydrogel	−0.9−0.1 V	202.79 mAh g^−1^ at 0.5 A g^−1^	10 000 (74.3%)	[207]
V_2_CT_x_ MXene	Ag/AgCl (Saturated)	Activated carbon	0.5 M NH_4_OAc	−1−(−0.1) V	115.9 mAh g^−1^ at 1 A g^−1^	5000 (100%)	[208]
M_o_S_2_@PANI	Ag/AgCl	Platinum	1 M NH_4_Cl	−0.6−0.4 V	450 F g^−1^ at 1 A g^−1^	5000 (86.3%)	[215]
PANI/PBAs	Ag/AgCl	Graphite rod	1 M (NH_4_)_2_SO_4_	−0.2−1 V	92.5 mAh g^−1^ at 0.1 A g^−1^	200 (93%)	[216]
VO_x_@PPy	SCE	–	0.5 M NH_4_OAc	−0.9−0 V	195.36 mAh g^−1^ at 0.2 A g^−1^	2000 (85%)	[33]
PANI‐intercalated V_2_O_5_	SCE	Platinum	0.5 M (NH_4_)_2_SO_4_	−0.5−0.9 V	307 mAh g^−1^ at 0.5 A g^−1^	100 (42%)	[47]
PANI‐intercalated V_2_O_5_	Ag/AgCl	Graphite rod	0.5 M (NH_4_)_2_SO_4_	−0.5−1 V	192.5 mAh g^−1^ at 1 A g^−1^	100 (98%)	[218]
KVO/PANI	Ag/AgCl	Carbon rod	1 M PVA/NH_4_Cl gel	−0.2−0.9 V	340 F g^−1^ at 0.5 A g^−1^	–	[220]

molality (m) = mol kg^−1^.

**Table 2:** The comparison of various AIBs based on organic electrodes in full cells.

Cathode	Anode	Electrolyte	Out voltage	Capacity	Cycle life (retention)	Energy/Power density [Wh Kg^−1^/W kg^−1^]	Refs.
(NH_4_)_1.47_Ni[Fe(CN)_6_]_0.88_	PTCDI	1 M (NH_4_)_2_SO_4_	0‐1.9 V	41 mAh g^−1^ at 0.06 A g^−1^	1000 (67%)	≈43/‐	[35]
NH_4_·Fe_4_[Fe(CN)_6_]_3_	PTCDI	Saturated (NH_4_)_2_SO_4_	0‐1.7 V	54.3 mAh g^−1^ at 1 C (mass of cathode)	300 (89.8%)	–	[50]
Mn_3_Al_1_‐LDH	PTCDI	0.5 M (NH_4_)_2_SO_4_	0‐1.7 V	57.7 mAh g^−1^ at 0.1 A g^−1^	100 (92%)	45.8/163.5	[46]
Od‐NHVO	PTCDI	1 M NH_4_Cl/PVA	0‐2.0 V	539 mF cm^−2^ at 1 mA cm^−2^	7000 (77%)	3 Wh m^−2^/10 W cm^−2^	[94]
N‐CuHCF	PTCDI	5.8 m (NH_4_)_2_SO_4_	0.4‐2.0 V	48.2 mAh g^−1^ at 5 C (mass of cathode)	1000 (≈72%)	–	[92]
(NH_4_)_1.85_Fe_0.33_Mn_0.67_[Fe(CN)_6_]_0.98_·0.77H_2_O	PTCDI	21 m NH_4_TFSI	0‐2.15 V	50.8 mAh g^−1^ at 0.04 A g^−1^	4000 (72.3%)	55.5/3600	[95]
FeMnHCF	PTCDI	24 m NH_4_CF_3_SO_3_	0‐2.5 V	123.8 mAh g^−1^ at 0.5 A g^−1^	3000 (67%)	≈71/‐	[98]
(NH_4_)_1.81_Fe_0.28_Mn_0.72_[Fe(CN)_6_]_0.96_·0.85H_2_O	PTCDI	2.5 m NH_4_OTF in succinonitrile	0‐2.25 V	116 mAh g^−1^ at 0.5 A g^−1^ (mass of cathode)	10 000 (73.9%)	65/600	[69]
VOPO_4_·2H_2_O	PTCDI	2 M NH_4_OTf in acetonitrile	0‐1.6 V	55 mAh g^−1^ at 0.1 A g^−1^	300 (‐)	–	[104]
CuHCF	PTCDI@MXene	2 m NH_4_OTf@ sucrose	0‐2.2 V	41 mAh g^−1^ at 0.2 A g^−1^	10 000 (80%) (−20 °C)	41.5/‐	[100]
CuHCF	PTCDI	1 M NH_4_OAc with EG	0‐2.0 V	70.4 mAh g^−1^ at 0.3 A g^−1^	500 (82.8%)	63.1/262.7 (mass of cathode)	[101]
FeHCF	PTCDI	1 m NH_4_OAc with EG	0‐1.6 V	95 mAh g^−1^ at 0.24 A g^−1^	1950 (77%)	–	[60]
KMnHCF	PTCDI	1 M NH_4_TFSI in TEGDME	0‐2.4 V	45 mAh g^−1^ at 0.03 A g^−1^	70 (‐)	‐	[93]
Graphite	PTCDI	1 m NH_4_PF_6_/ ADN‐EMC	0.75‐2.75 V	107.9 mAh g^−1^ at 2 C	1000 (88%)	200/‐ (mass of cathode)	[70]
MnO_2_	PTCDA	1 M (NH_4_)_2_SO_4_	0‐1.9 V	≈100 mAh g^−1^ at 0.1 A g^−1^ (mass of cathode)	4000 (51.8%)	68.2/8211.6	[111]
Carbon cloth	PTCDA	7 m NH_4_Br + 1 m TPABr	0.6‐1.6 V	118 mAh g^−1^ at 0.5 A g^−1^ (mass of anode)	2000 (79.4%)	113/‐	[112]
N‐NiHCF	PTCDA	1 m NH_4_OAc with 0.4% PEO	0‐1.8 V	44.05 mAh g^−1^ at 0.2 A g^−1^ (mass of cathode)	1000 (98.4%)	–	[118]
Ni‐APW	ALO	1 M (NH_4_)_2_SO_4_	0.2‐1.6 V	128.7 mAh g^−1^ at 0.5 A g^−1^ (mass of anode)	10 000 (no attenuation)	102.4/5055	[121]
PTMA	PI	1 M (NH_4_)_2_SO_4_	0‐1.9 V	136.5 mAh g^−1^ at 0.5 A g^−1^	10 000 (84.6%)	51.3/15800	[128]
(NH_4_)_2_V_6_O_16_∙1.5 H_2_O/C	PNTCDA	1 M NH_4_Cl	0‐1.4 V	54.1 mAh g^−1^ at 0.1 A g^−1^	300 (96.1%)	–	[130]
PANI/CNF	PI/NDC/CNT	1 M (NH_4_)_2_SO_4_	0‐1.9 V	136.7 mAh g^−1^ at 0.5 A g^−1^	2000 (‐)	114.3/18600	[131]
Ni‐APW	PNNI	1 M NH_4_OAc	0‐1.8 V	44.4 mAh g^−1^ at 0.3 A g^−1^	10 000 (close to 100%)	68.7/383.8	[132]
NiHCF@CNTs	Poly(1,5‐ NAPD)	19 m NH_4_OAc	0.4‐1.5 V	143 mAh g^−1^ at 1 A g^−1^ (mass of anode)	500 (88.5%)	31.8/2266	[138]
NH_4_V_3_O_8_·2.9H_2_O	PANI	1 M (NH_4_)_2_SO_4_	0‐1 V	121 mAh g^−1^ at 0.1 A g^−1^	400 (95%)	–	[126]
CF@NH_4_V_4_O_10_	CF@PANI	1 M (NH_4_)_2_SO_4_	0‐1 V	167 mAh g^−1^ at 0.1 A g^−1^	1000 (73.3%)	–	[160]
CuHCF	PANI	2 M NH_4_NO_3_	0‐0.9 V	55.3 mAh g^−1^ at 2 A g^−1^	1240 (74.3%)	–	[161]
PA@POFA@C	NH_4_V_4_O_10_	1 M NH_4_OAc	0‐1.5 V	38 mAh g^−1^ at 0.2 A g^−1^	1000 (74%)	13.89/72.87	[149]
PANI	VO_2_·xH_2_O	1 M (NH_4_)_2_SO_4_	0‐1.4 V	216 mAh g^−1^ at 0.1 A g^−1^ (mass of anode)	1000 (80%)	‐/4540	[163]
PPy	PANI	19 m NH_4_OAc	0‐1 V	78.4 mAh g^−1^ at 0.1 A g^−1^ (25 °C)	200 (86.72%)	–	[164]
CuHCF	HATP‐PT COF	2 m NH_4_OTf@ sucrose	0‐2 V	30.4 mAh g^−1^ at 0.4 A g^−1^	20 000 (89%)	–	[84]
AC	PTCDI /rGO	32 m NH_4_OAc	0‐1.9 V	15.3 mAh g^−1^ at 1 A g^−1^	3000 (74%)	12.9/827	[197]
M_o_S_2_@PANI	M_o_S_2_@PANI	PVA‐NH_4_Cl gel	0‐1.4 V	219.8 F g^−1^ at 1 A g^−1^	10 000 (80.5%)	59.8/725.4	[215]
PANI/PBAs	PI@MXene	1 M (NH_4_)_2_SO_4_	0‐1.8 V	52.5 mAh g^−1^ at 1 A g^−1^	200 (83%)	–	[216]
NI‐Co LDH‐A	VOx@PPy	0.5 M NH_4_OAc	0‐1.7 V	191.2 mAh g^−1^ at 0.2 A g^−1^	3000 (81.1%)	74.1/75.6	[33]
KVO/PANI	AC	1 M PVA/NH_4_Cl gel	0‐1.6 V	376 mF cm^−2^ at 1 mA cm^−2^	10 000 (61%)	31.8/47.6	[220]

## 3 Summary and Outlook

### 3.1 Structural Design of Organic Electrode Materials

AAIBs offer a wide range of advantages, rendering them a compelling choice for high performance energy storage devices. The small hydrated ionic radius of the NH_4_^+^ ions involved contributes to fast ion diffusion, enhancing the rate performance of these batteries. Safety and environmental benignity are also key benefits, AAIBs use non‐toxic, eco‐friendly electrolytes that are less corrosive and possess lower potentials for HER compared to acidic electrolytes. The electrochemical performance of AAIBs is highly dependent on the selection of electrode materials, with organic compounds emerging as particularly promising due to their customizable properties. The fine‐tuning of these materials hinges on a deep understanding of their redox chemistry, especially how electron‐donating and electron‐withdrawing groups impact the HOMO, LUMO, and the reduction potential. Electron‐donating groups (EDGs), such as hydroxyl (‐OH), methoxy (‐OCH₃), and amino (‐NH₂) groups, elevate the electron density within the conjugated system of organic molecules, thereby increasing the HOMO level. This enhancement facilitates easier oxidation of organic materials, reducing the oxidation potential and potentially boosting the overall battery voltage. Conversely, electron‐withdrawing groups, like nitro (‐NO₂), cyano (‐CN), carboxyl (‐COOH), and halogens (e.g., chlorine ‐Cl, bromine ‐Br), reduce the electron density, resulting in a lower LUMO level. This adjustment increases the susceptibility of organic materials to reduction, elevating the reduction potential. While this typically results in a decrease in voltage, it also enhances the material's stability against over‐reduction. For instance, novel compounds featuring ‐NO₂ groups present intriguing possibilities as potential candidates.^[^ ^223^, ^224^ ^]^ While a smaller bandgap can improve reaction kinetics, it also increases the organic material's susceptibility to side reactions and decreases stability. An excessively narrow bandgap might trigger unwanted charge carrier recombination or degradation processes, ultimately shortening AAIBs’ cycle life and reducing the overall efficiency. Therefore, the challenge lies in finding an optimal bandgap that is narrow enough to facilitate rapid reaction kinetics without compromising the organic material's stability or inducing side reactions. Organic electrode materials offer the advantage of being tunable through the incorporation of various functional groups, allowing for the careful adjustment of the bandgap. This customization enables the optimization of both reaction kinetics and overall battery performance. A key future direction in synthesizing redox‐active organic molecules and their polymers is to reduce synthesis steps and use more environmentally friendly or solvent‐free conditions.^[^ ^225^ ^]^ Prioritizing sustainability and cost‐effectiveness is crucial. Although bipolar organic molecular systems hold promise for AAIBs, their electrochemical performance is still lacking, highlighting the need for new bipolar polymers or small molecules with advanced chemical designs.^[^ ^226^, ^227^ ^]^ Surface and morphology engineering, interlayer modification, heterostructures, and compositional changes such as heteroatom doping are advanced strategies used in the design of organic electrode materials to enhance the performance of AAIBs. For instance, creating a hierarchical porous structure (micro‐, meso‐, and macropores) in electrode materials allows for better electrolyte penetration and faster ion transport and electron conduction. Designing 3D architectures^[^ ^228^ ^]^ or increasing the interlayer spacing can facilitate the insertion and extraction of large ions such as NH_4_^+^, thus improving the battery's capacity and rate performance. Encapsulation of organic materials with a protective barrier or matrix can significantly enhance the cycle life of AAIBs by reducing the loss of active organic materials and preventing degradation reactions that occur during repeated charging and discharging cycles. Self‐healing organic materials,^[^ ^76^ ^]^ which can autonomously repair damage, represent a promising advancement with the potential to significantly extend the operational lifespan of organic electrodes in AAIBs.

### 3.2 Characterization Techniques

Integrating in situ and ex situ techniques provides a holistic view of the behavior of organic materials during operation and after extended cycling.^[^ ^229^ ^]^ In situ techniques allow for real‐time monitoring of dynamic changes in organic materials, capturing how they respond under actual working conditions. Meanwhile, ex situ analysis offers a detailed post‐mortem examination, revealing structural, chemical, and morphological alterations that occur over time. Together, these approaches deliver a more complete understanding of the organic material's performance, degradation mechanisms, and long‐term stability. The combination plays a crucial role in elucidating degradation mechanisms, identifying failure modes, and informing the development of more stable and efficient organic electrode materials for AAIBs. For example, SSNMR, differential electrochemical mass spectrometry, wide‐angle X‐ray scattering, and small‐angle neutron scattering are highly effective techniques for characterizing and analyzing organic materials across different scales. However, devising reliable and innovative means to conclusively identify and characterize the NH₄⁺ diffusion processes driven by electrostatic interactions, including hydrogen bonding, remains a significant challenge.

### 3.3 Electrolyte Optimization

To address the challenges posed by the narrow electrochemical working window of aqueous electrolytes, especially in organic batteries and systems like AAIBs, researchers are actively developing new electrolyte formulations and additives. These advancements focus on enhancing ionic transport, broadening the working window, optimizing the electrode/electrolyte interface, and mitigating problems such as water activity and freezing in diluted electrolytes. One promising approach is the use of hydrogel electrolytes, which offer unique properties such as flexibility, high ionic conductivity, and tunable mechanical strength. Various methods for preparing hydrogel electrolytes, including cross‐linking, polymerization, and the incorporation of nanomaterials,^[^ ^230^, ^231^, ^232^ ^]^ are being explored to further improve the performance and stability of AAIBs. The use of flexible, conductive substrates facilitates the accommodation of the expansion and contraction experienced by organic materials during charge and discharge cycles. This flexibility helps to reduce mechanical stress, thereby enhancing the durability and extending the lifespan of the organic materials. To successfully integrate water‐in‐salt electrolytes into AAIBs, it is essential to carefully choose salts that strike an optimal balance between ionic conductivity, viscosity, and electrochemical stability. Selecting the right salts ensures that the electrolyte performs efficiently, maintains low resistance, and supports stable operation over a wide range of conditions, for example low temperatures. Enhancing the stability of the electrode/electrolyte interface is critical to prevent unwanted side reactions and corrosion in AAIBs. Common strategies include surface coatings to protect the electrode, electrolyte additives to stabilize the interface, and the development of advanced organic electrode materials. For instance, adding specific additives to the electrolyte can help stabilize the interface by altering the chemical environment and reducing the occurrence of undesirable reactions.^[^ ^118^, ^233^ ^]^ However, achieving an optimal balance between improving electrolyte conductivity and preserving a broad electrochemical stability window, particularly when additives are introduced to disrupt water‐water hydrogen bonding networks, remains a complex and unresolved challenge. Grasping how ammonium salts decompose thermally and how this affects the performance and safety of AAIBs is vital for developing durable and efficient battery technologies. Implementing robust thermal management, choosing materials with high stability, and establishing thorough safety protocols are crucial to addressing the challenges presented by ammonium salt decomposition. These approaches are necessary to maintain the reliability and extend the lifespan of AAIBs, especially in demanding or high‐temperature environments.

### 3.4 Advanced Electrode Fabrication Techniques

**Figure 14:**
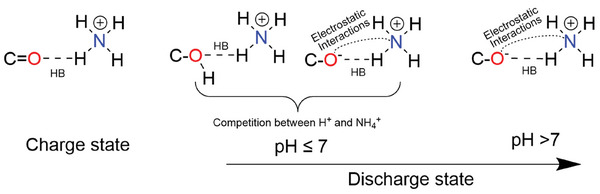
The possible mechanisms of interaction between C═O and NH_4_^+^ during charge/discharge under different pH conditions, which remain speculative and require further experimental validation.

The competition between NH_4_^+^ and H^+^ at the electrode/electrolyte interface is a key factor influencing the efficiency and reversibility of the energy storage process in AAIBs. **Figure** **14** illustrates the potential mechanisms of interaction between C═O and NH_4_^+^ under different pH conditions. In the charged state, this interaction is primarily governed by hydrogen bonding. During the discharge process, both electrostatic interactions and hydrogen bonding between C‐O⁻ and NH_4_^+^ can coexist.^[^ ^83^ ^]^ For instance, under neutral or acidic conditions (pH ≤ 7), the coexistence of NH_4_^+^ and H^+^ can result in competing electrochemical reactions, potentially disrupting ion transport and compromising the stability of electrode materials over repeated charge and discharge cycles.^[^ ^132^ ^]^ Achieving an optimal balance between these ions is essential for improving energy density and extending the cycling stability of AAIBs. Advanced fabrication techniques, including layer‐by‐layer deposition and solvent annealing, which precisely control the morphology and uniformity of organic electrode materials, can greatly enhance cycling stability. 4D printing in the realm of organic‐based AAIBs utilizes smart materials that can self‐assemble or transform their configuration over time in response to external stimuli.^[^ ^234^ ^]^ These materials, such as shape‐memory polymers and responsive hydrogels, are combined with organic electroactive components to produce batteries with adaptive properties. This innovative approach results in batteries that are not only flexible and lightweight but also capable of self‐healing and self‐repairing, significantly extending their lifespan and enhancing their performance. The integration of 4D printing with organic battery technology allows for the creation of batteries that can dynamically modify their internal structures to achieve optimal performance. For instance, the internal arrangement of electrodes or electrolytes can be reconfigured to minimize internal resistance or enhance ion transport, thereby boosting the battery's energy density and power output.

### 3.5 Theoretical Calculations

DFT calculations, molecular dynamics (MD) simulations, and quantum chemical calculations are vital computational methods for advancing the design and understanding of AAIBs. DFT calculations can provide detailed insights into the electronic structures and energetics of organic electrode materials, enabling the prediction of their electrochemical behavior and stability in aqueous environments. MD simulations are particularly useful for modeling the movement of ions and water molecules in the electrolyte, revealing critical details about ionic conductivity, diffusion pathways, and solvation dynamics. Quantum chemical calculations complement these approaches by offering precise descriptions of molecular interactions and the electronic states involved in redox reactions. Together, these computational techniques allow for a comprehensive exploration of the complex mechanisms in AAIBs, guiding the development of novel organic electrode materials and optimizing their performance for safer, more stable, and sustainable energy storage solutions.

### 3.6 Toward Large‐Scale Applications

Organic‐based AAIBs offer significant potential as eco‐friendly and sustainable energy storage solutions. Despite these advantages, their relatively low specific capacity and energy density pose substantial challenges to their widespread use, particularly in applications where reducing size and weight is essential, such as in electric vehicles and portable electronics. Addressing these limitations will require ongoing research and development to enhance the energy density of these batteries. Potential strategies to achieve this include developing novel materials, designing more efficient battery architectures, or creating hybrid systems that combine organic materials with alternative components to optimize performance. It is crucial to maintain optimal electrochemical performance in AAIBs as the mass loading of organic electrodes is scaled up to commercial levels, given that current systems often have relatively low mass loading.^[^ ^235^ ^]^ Increasing the mass loading of active organic materials is a critical step toward boosting the commercial viability of AAIBs. Maintaining these performance characteristics is crucial for effectively transitioning from laboratory‐scale development to large‐scale industrial production, thereby facilitating successful market adoption and practical deployment.

## Conflict of Interest

The authors declare no conflict of interest.
